# Proteomic and bioinformatic pipeline to screen the ligands of *S*. *pneumoniae* interacting with human brain microvascular endothelial cells

**DOI:** 10.1038/s41598-018-23485-1

**Published:** 2018-03-27

**Authors:** Irene Jiménez-Munguía, Lucia Pulzova, Evelina Kanova, Zuzana Tomeckova, Petra Majerova, Katarina Bhide, Lubos Comor, Ivana Sirochmanova, Andrej Kovac, Mangesh Bhide

**Affiliations:** 1 Laboratory of Biomedical Microbiology and Immunology, University of Veterinary Medicine and Pharmacy in Kosice, Kosice, Slovak Republic; 2 Institute of Neuroimmunology of Slovak Academy of Sciences, Bratislava, Slovak Republic

## Abstract

The mechanisms by which *Streptococcus pneumoniae* penetrates the blood-brain barrier (BBB), reach the CNS and causes meningitis are not fully understood. Adhesion of bacterial cells on the brain microvascular endothelial cells (BMECs), mediated through protein-protein interactions, is one of the crucial steps in translocation of bacteria across BBB. In this work, we proposed a systematic workflow for identification of cell wall associated ligands of pneumococcus that might adhere to the human BMECs. The proteome of *S*. *pneumoniae* was biotinylated and incubated with BMECs. Interacting proteins were recovered by affinity purification and identified by data independent acquisition (DIA). A total of 44 proteins were identified from which 22 were found to be surface-exposed. Based on the subcellular location, ontology, protein interactive analysis and literature review, five ligands (adhesion lipoprotein, endo-β-N-acetylglucosaminidase, PhtA and two hypothetical proteins, Spr0777 and Spr1730) were selected to validate experimentally (ELISA and immunocytochemistry) the ligand-BMECs interaction. In this study, we proposed a high-throughput approach to generate a dataset of plausible bacterial ligands followed by systematic bioinformatics pipeline to categorize the protein candidates for experimental validation. The approach proposed here could contribute in the fast and reliable screening of ligands that interact with host cells.

## Introduction

*Streptococcus pneumoniae* is one of the longest-known pathogens responsible for sepsis, meningitis and pneumonia. Meningitis-causing *S*. *pneumoniae* can cross the blood-brain barrier (BBB) as live bacteria via transcellular mechanism and then multiply inside the central nervous system (CNS)^1^. The human BBB is a structural and functional barrier formed by brain microvascular endothelial cells (BMEC). Together with glia, astrocytes, pericytes and smooth muscle cells they form neurovascular unit (NVU). Although, this is the most sophisticated barrier that prevents entry of toxins, harmful metabolites and infectious agents into the CNS, several pathogens possess mechanisms of its crossing^2^.

The receptor-mediated adhesion is considered as a key event in the process of invasion of pathogen across BBB. Binding of bacterial ligands to the specific host cell receptors (ligand-receptor interactions) may lead to signal transduction resulting in tight bacterial attachment to the host cells, internalization by the cells or alteration in the permeability of BBB^3,4^. Surface-displayed proteins (pathogen ligands) are immediate emissaries for contact with the endothelial lining. For example, cell wall proteins of pneumococcus readily activates the platelet-activating factor receptor (PAF) on BMEC^5,6^. Hitherto, the precise mechanisms by which surface proteins of *S*. *pneumoniae* interact to their counterpart receptors on BMECs and subsequently help pneumococcus to penetrate the BBB are not fully discovered. Plethora of pneumococcal proteins are theoretically predicted to have potential of surface exposure (557 candidates, predicted based on locateP,^7^), however, only few are studied for their role in the invasion and development of meningitis^8–11^.

The analysis of bacterial surface-associated proteins is challenging because of their physico-chemical properties and autolysis or fratricide committed during bacterial growth^12–14^. Hitherto, classical gel-based proteomic approaches have been applied for analysis of surface-exposed bacterial ligands and their receptors on the host cells^15–17^. However, these techniques have several limitations e.g. it is difficult to resolve complex proteins with highly hydrophobic moieties^18^, they are suboptimal for study of membrane-embedded and low-abundant proteins, and techniques are laborious, time consuming and extremely low-throughput. Furthermore, gel-based techniques become bottle neck in the ligand-receptor interactions experiments especially when biological material is scanty (e.g. primary cells from brain microvasculature). Due to these limitations, many of interacting ligands or receptors involved in meningitis could remain undetected.

Hitherto, the classical *in vivo* approaches have been used to study the role of certain molecules such as lipoteichoic acid, peptidoglycan or choline binding proteins in meningitis^19–21^. Development of robust and high-throughput experimental approaches for studying interactions between pathogens and cells derived from the NVU is demanded for better understanding of the basic principles of neuroinvasion of *S*. *pneumoniae*. For example, genome sequencing, a high-throughput technique, has been applied for meningitis-causing microbes (e.g. *E*. *coli*, *N*. *meningitidis*, *M*. *tuberculosis*, etc.) to elucidate microbial basis of translocation of the blood-brain barrier^22–25^. High-throughput gel-free proteomics has contributed in the analysis of entire proteomes of pathogenic bacteria yielding in the identification of low abundant, hydrophobic or complex proteins^13^. However, to our knowledge the role surface-exposed proteins of pneumococci in meningeal infection has not been addressed yet with robust proteomic approaches.

Protein labeling in combination with high-throughput analysis has contributed in the elucidation of several molecular mechanisms. Biological research often requires use of molecular labels that attach to proteins of interest and facilitate their detection or purification. Nowadays, multiples types of labels (e.g. biotin, active site probes, enzyme conjugates, etc.) are available, however it is necessary to choose carefully labeling strategy for each study. Labeling with a thiol-cleavable amine-reactive biotinylation reagent has been successfully applied for the identification of interacting molecules easily recovered with high affinity NeutrAvidin®^26^. Biotinylation of proteins exposed on the bacterial surface requires the presence of free amino groups to the external milieu, however for pathogens such as *S*. *pneumoniae*, which possesses a thick polysaccharide capsule of which only few proteins protrude, biotinylation of the entire proteome (cell lysate) could contribute to the identification of maximum number of ligands than biotinylation of intact bacteria.

High-throughput proteomic approaches (e.g. whole proteome labeling followed by identification of candidates of interest with LC-MS), generates huge amount of data and list of interacting proteins, which could drive into a complicated data processing. Once a list of interacting partners is obtained these protein-protein interactions are typically visualized within a functional network, which helps to identify the underlying biology in host–pathogen interactions. Common resources for network visualization include STRING and Cytoscape. Although these tools have become fairly standard for the proteomics field, they have been employed to an even lesser extent in bacterial infections. Another limitation of immuno-affinity purification or biotinylated proteome based pull down assay coupled to LC-MS is the presence of non-specifically interacting proteins in MS datasets that co-purify with the protein of interest. It is necessary to filter the false-positive protein candidates with bioinformatic algorithm. Although such algorithms are available (e.g. SAINT - http://saint-apms.sourceforge.net/; CRAPome - http://crapome.org), they are not sufficient for filtering the bacterial ligands. Furthermore, due to the diversity of the bacterial cell wall structure (e.g. capsulated, non-capsulated, presence or absence of teichoic acid, etc.), custom tailored algorithms are more effective to retrieve biological relevance from MS dataset. It is possible to set up such algorithm by using tens of existing bioinformatic tools available freely (e.g. to predict subcellular location, antigenicity, gene ontology, etc.).

In the present study, we attempted to identify pneumococcal proteins that may interact with the cells of NVU. First, we biotinylated the whole protein content of *S*. *pneumoniae*, recovered pneumococcal proteins interacting with BMECs and identified the potential ligands by SWATH-MS. Further, we established a systematic bioinformatics workflow to analyze the data derived from SWATH-MS using freely available tools and selected potential ligands for confirmation of interaction with BMECs. We were able to validate affinity of selected bacterial ligands (Adhesion lipoprotein, Spr0996; PhtA, Spr1061; endo-β-N-acetylglucosaminidase, Spr0440; and two hypothetical proteins, Spr0777 and Spr1730) to BMECs, which may have a crucial role in the commencement of neuroinvasion. This approach could also be used for other pathogens to unfold protein-protein interactions.

## Results

### Ligands of *S*. *pneumoniae* plausibly interacting with human BMEC

**Figure 1:**
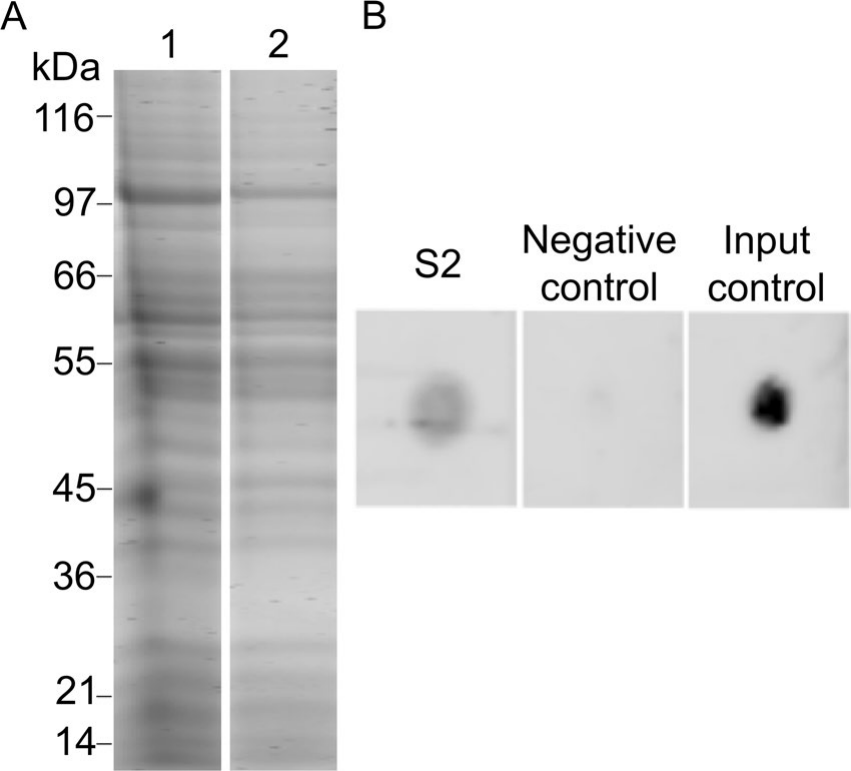
Biotin labeling of proteome of pneumococci and confirmation of the presence of biotinylated proteins bound to human BMEC. Panel A shows protein labeling. Lane 1, protein extract of pneumococci prior to biotinylation separated on SDS-PAGE. Lane 2, biotinylated proteins were incubated on NeutrAvidin capture beads, eluted with 50 mM DTT and separated on SDS-PAGE. Lane 1 and 2 were cropped from original image obtained after PAGE (see Supplementary Figure 5). Note that the gel depicted here is representative of four replicates. Panel B shows the presence of biotinylated proteins in S2.Protein extract of BMEC obtained after incubation of biotinylated proteins of pneumococci with BMEC was spotted on the membrane and detected with IRdye®800 Streptavidin (S2) in dot blot. Total protein extract of human BMECs was spotted on membrane and incubated with IRdye®800 Streptavidin (negative control); Biotinylated proteins of pneumococci were spotted on membrane and detected with IRdye®800 Streptavidin (input control). (**B**) was created by combining cropped fragments from two membranes (see Supplementary Figure 6).

Receptor-mediated binding to the plasma membrane of endothelial cells can facilitate the pneumococcal translocation through the BBB. To identify potential ligands of pneumococci in receptor-mediated interaction, we biotinylated pneumococcal proteome for subsequent binding with human BMEC. Prior to perform the ligand-BMEC interaction assay, we corroborated the biotinylation of the pneumococcal protein species by capturing them on NeutrAvidin capture beads. Maximum number of protein species were observed in the profile of labeled proteins (Fig. 1A, lane 2), when compared to the profile of non-labeled proteins (Fig. 1A, lane 1).

After incubation of biotinylated proteome with the host cells, non-interacting proteins were removed and interacted proteins were recovered in the protein fraction (S2). Presence of biotinylated proteins in S2 was confirmed in dot blot (Fig. 1B). Subsequently biotinylated proteins were recovered from protein fraction (S2) with NeutrAvidin® capture beads to proceed with protein identification using SWATH-MS. The MS-based shotgun proteomic tool succeeded in identification of challenging proteins. Protein identification yielded forty-four pneumococcal proteins as potential ligands (see Supplementary Dataset 1).

**Figure 2:**
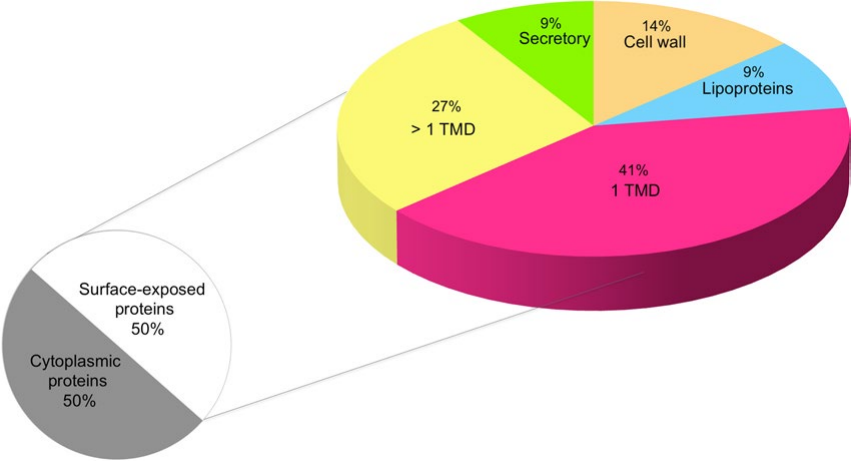
Subcellular location of protein predicted by bioinformatics workflow. Subcellular predictions and feature-based algorithms were used to segregate surface-exposed proteins. Surface proteins were further sub-categorized into secretory, cell wall attached proteins with a LPxTG domain, proteins containing a signal sequence SP-II (Lipoproteins), proteins embedded in the cell membrane with one (1TMD) or more (>1TMD) transmembrane domain/s.

Since the surface-exposed proteins are crucial in adhesion of pathogens on the host cells (BMECs in our study), we established a systematic bioinformatics pipeline to remove proteins (in this case cytoplasmic proteins) with the minimal potential to interact with BMECs. Following this pipeline, a set of 44 proteins was apportioned into 22 cytoplasmic and 22 surface-exposed proteins (locateP, PSortb). Surface-exposed were further categorized into, 3 cell wall-attached proteins, 2 lipoproteins (LipoP), 2 secreted (SignalP), and 15 membrane proteins (TMHMM, HMMtop). Among 15 membrane proteins, 6 were embedded by more than one transmembrane domain (TMD) and 9 by only one (Fig. 2, Table 1).

**Table 1:** Surface-exposed proteins of *S*. *pneumoniae* identified in our study by SWATH-MS.

No.	Entry	Protein name	Locus	Location	Bioinformatic tools
1	Q8CZ52	LPXTG-motif cell wall anchor protein	spr0440	Cell wall	A, B
2	Q8DRK2	Uncharacterized protein	spr0075	Cell wall	A, B
3	Q8CYC9	Plasmin and fibronectin-binding protein A	spr1652	Cell wall	A, B
4	Q8CYI8	Uncharacterized protein	spr1403	Membrane (1TMD)	A, B, E, F
5	Q8DPQ2	Pneumococcal histidine triad protein A (phtA)	spr1061	Membrane (1TMD)	A, B, E, F
6	P67294	UPF0154 protein spr1697	spr1697	Membrane (1TMD)	A, B, E, F
7	Q8DPY9	Pneumococcal vaccine antigen A	spr0930	Membrane (1TMD)	A, B, E, F
8	P0A4G3	Manganese ABC transporter substrate-binding lipoprotein (Pneumococcal surface adhesin A)	spr1494	Membrane (1TMD)	A, B, E, F
9	Q8CWR4	Histidine Motif-Containing protein	spr1060	Membrane (1TMD)	A, B, E, F
10	Q8DQW4	Uncharacterized protein	spr0466	Membrane (1TMD)	A, B, E, F
11	Q8DRI0	Surface protein pspA	spr0121	Membrane (1TMD)	A, B, E, F
12	Q8DR59	Penicillin-binding protein 1 A (PBP-1A) (Exported protein 2)	spr0329	Membrane (1TMD)	A, B, E, F
13	Q8DNE1	Membrane protein insertase YidC 1 (Foldase YidC 1) (Membrane integrase YidC 1) (Membrane protein YidC 1)	spr1790	Membrane (>1TMD)	A, B, E, F
14	Q8CYB8	Hypothetical protein	spr1730	Membrane (>1TMD)	A, B, E, F
15	Q8DQ98	Hypothetical protein	spr0777	Membrane (>1TMD)	A, B, E, F
16	Q8DQ02	MesH protein	spr0916	Membrane (>1TMD)	A, B, E, F
17	Q59947	Immunoglobulin A1 protease (IgA1 protease) (IgA-specific zinc metalloproteinase)	spr1042	Membrane (>1TMD)	A, B, E, F
18	Q8DQN5	Zinc metalloprotease ZmpB	spr0581	Membrane (>1TMD)	A, B, E, F
19	Q8DN05	Choline binding protein A	spr1995	Secretory	A, B, D
20	Q7ZAK5	Pneumolysin (Thiol-activated cytolysin)	spr1739	Secretory	A, B, D
21	P59214	Maltose/maltodextrin-binding protein	spr1918	Lipoprotein	A, B, C
22	Q8DQ09	Adhesion lipoprotein	spr0906	Lipoprotein	A, B, C

Categories for the subcellular location were established according to LocateP. Membrane proteins were categorized as: 1TMD, proteins containing one transmembrane domain; and >1TMD, proteins anchored to plasma membrane by more than one transmembrane domain. Secretory and lipoproteins were those proteins containing a SP-I type and SP-II type signal peptides, respectively. Finally, cell wall proteins were those possessing a LPxTG motif. Bioinformatic tools are indicated as follows: A, LocateP; B, PSortb; C, Lipo P; D, Signal P: E, TMHMM; and F, HMMtop.

It is noteworthy that, 4 proteins were identified that bind to collagen or tissue culture plastic (In absence of BMECs in tissue culture flask, negative control, see Supplementary Dataset 1). One of those four candidates, Spr1403 - uncharacterized protein, was also present in the list of proteins that may interact with BMECs. It is important to take great caution while predicting function of such candidate. Although we have included Spr1403 in downstream bioinformatics pipeline, we did not include it for further validation of protein-BMEC interaction with ELISA or immunocytochemistry.

### Detailed analysis of interacting proteins of *S*. *pneumoniae*

Surface-exposed proteins of pathogenic bacteria play a crucial role in the development of infections. Thus, only surface-exposed proteins were targeted further. Blast2GO analysis employed to retrieve protein annotations, revealed that 45% of proteins (10 out of 22 candidates) or their orthologs are non-annotated (neither in UNIPROT). The most representative functions retrieved from the GO annotations were cell adhesion, transmembrane transport and biosynthetic mechanisms (Biological process, Supplementary Figure 1A) and ion binding (Molecular function, Supplementary Figure 1B).

The ontology analysis revealed that Spr1790 plays role in the membrane organization and transport. Spr1697 and Spr1918 are associated with transport of solutes across lipid bilayer. Spr0329 binds penicillin and interact selectively as well as non-covalently with ions. Spr1042 and Spr0581 are peptidases. Spr1494 and Spr0906 play a role in adhesion, ion binding and transport, whereasSpr1739 is a lipid-binding protein (Supplementary Table 1).

Among the surface-exposed proteins Spr0440, Spr1403, Spr0075, Spr0466, Spr1730, and Spr0777 are uncharacterized proteins. Ontology analysis based on their identity with proteins from other related strains showed 99% identity in case of Spr0440 and Spr0075, with endo-β-N-acetylglucosaminidase (SP0498) and cell wall surface anchor protein (SP0082) of *S*. *pneumoniae* TIGR4, respectively. The later contains a single YSIRK and fibronectin-binding repeat SSURE domains.

BLAST2GO, however, failed to provide any relevant information for Spr1403, Spr1995, Spr0930, Spr1060, Spr1730, Spr0777, Spr1061, Spr0121, Spr1652 (Supplementary Table 1). The literature review performed for these nine proteins revealed that Spr1995 (choline binding protein A), Spr1061 (pneumococcal histidine triad protein A) and Spr1652 (plasmin and fibronectin-binding protein A) may have adhesion function (Supplementary Table 1). Choline binding protein A, pneumococcal vaccine antigen A, histidine motif-containing protein and pneumococcal histidine triad protein A are candidates in vaccine design. Protein spr1403 is used in serodiagnostics. Further, with literature review it is found that choline binding protein A, pneumococcal histidine triad protein A, surface protein pspA and plasmin and fibronectin-binding protein A involve in the biological processes such as colonization of respiratory tract or pathogenesis pneumonia (Supplementary Table 1).

The last part of the bioinformatics analyses, included in the proposed workflow, consisted *in silico* evaluation of protein interactive networks performed with The Search Tool for Retrieval of Interacting Genes (STRING). The analysis was carried to see possible associations among the subset containing surface-exposed proteins as well as other proteins present in the repository that could be important in the pathogenesis. From this analysis, we found two networks. The first network (Network 1, Supplementary Figure 2) consisted of well-characterized proteins (PspA, Ply, PspC, ZmpB, PsaA and Iga) of which at least 3 proteins are associated with meningitis (all proteins identified in our study, Supplementary Table 1). The second network (Network 2) comprised of 5 well-characterized ligands and one unknown candidate (Supplementary Figure 2).

### Selection of the protein candidates for validation of ligand-BMEC interaction

Once the surface-exposed proteins were categorized and the biological relevance was retrieved with bioinformatics workflow, 5 proteins were selected to confirm their binding with BMECs. Based on the subcellular location, we selected one cell wall protein (Spr0440), one lipoprotein (Spr0906), one membrane protein attached by one transmembrane domain (Spr1061) and two membrane proteins attached by more than one transmembrane domains (Spr0777 and Spr1730). Moreover, these proteins were considered as optimal candidates because of additional properties observed in our bioinformatics analyses as follows: Spr0440 was identified as a part of the protein interactive network (Network 2, Supplementary Figure 2) after STRING analysis; function of Spr1730 and Spr0777 is completely unknown; the Spr1061 is related to pathological processes; and the fifth candidate, Spr0906, is annotated as adhesin in GO analysis.

### Validation of the protein-protein interactions and ligand-BMEC interactions

**Figure 3:**
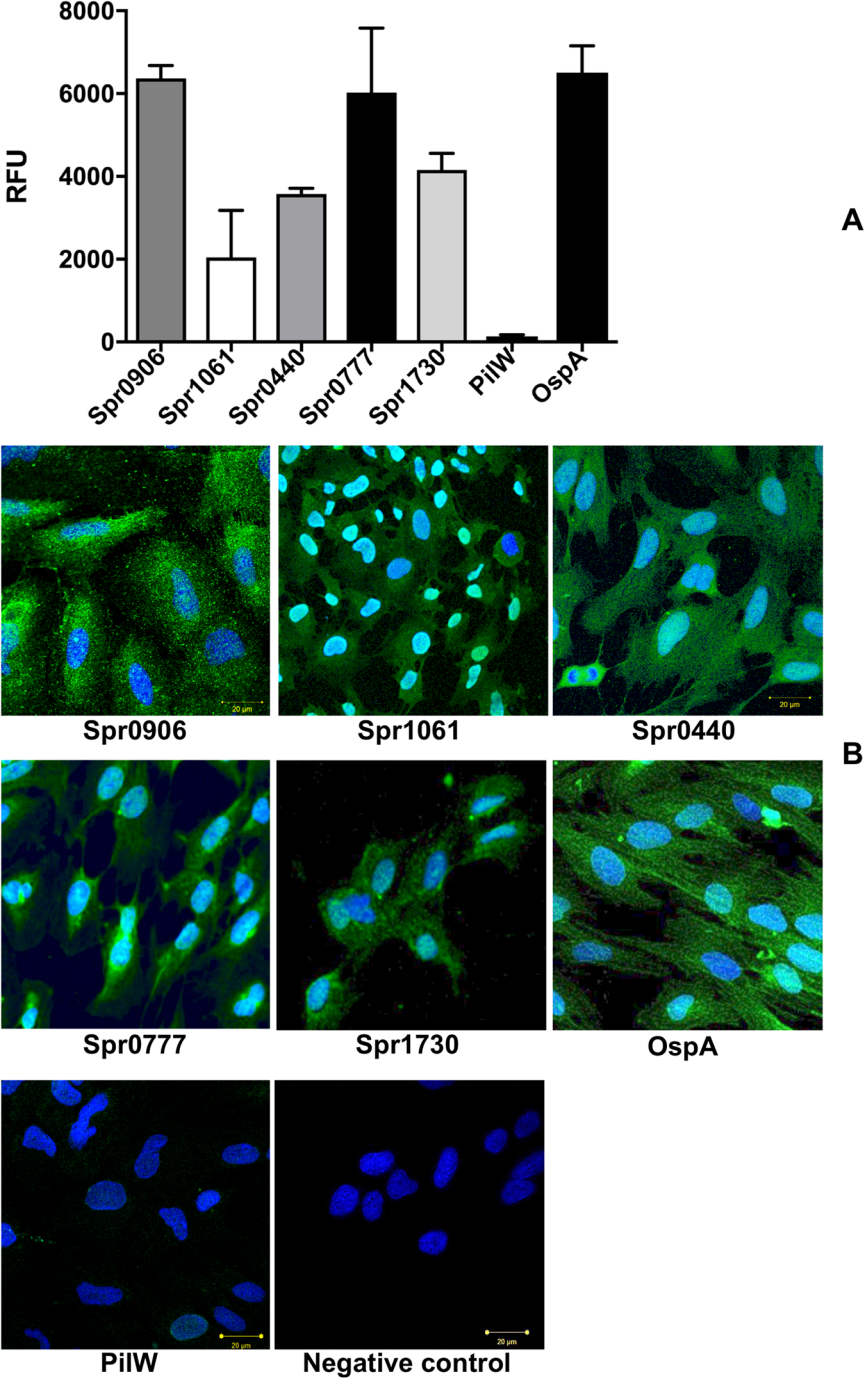
Confirmation of interaction between selected ligand candidates and protein of BMEC or cultured BMEC. Panel A: Semi-quantitative ELISA performed to confirm interaction of bacterial ligands with proteins of BMECs. Bar graph shows interaction of the selected potential ligands recombinantly produced tagged with GFP and protein extract from human BMEC. Interaction, performed in biological triplicate, was detected with anti-GFP antibody and is represented in Relative Fluorescence Units (RFU). Each RFU value for ligands is presented after subtraction of background of negative control as follows: RFU of ligand – RFU of negative control (no ligand was added; the well contained BMEC protein, secondary antibody and substrate only). Panel B. Interaction of selected pneumococcal proteins with cultured BMECs. Nuclei are stained with DAPI. Negative control – no recombinant ligand was included in assay. Scale bars – 20 µm. In both panels: Spr0906 – Adhesion lipoprotein, Spr1061 - Pneumococcal histidine triad protein A, Spr0440 - LPXTG-motif cell wall anchor protein, Spr0777 - Hypothetical protein, Spr1730 - Hypothetical protein, PilW– protein of *Francisella* known to not interact with BMECs. OspA - protein of neuroinvasive *Borrelia* well known to interact with BMECs.

In order to confirm binding ability of pneumococcal surface ligands to human BMEC, we generated five recombinant ligands (selected above) encompassing the extracellular domain (Table 2). In subcloning presence of the genes of interest in transformants was detected with vector specific primers (Supplementary Figure 3). Purity of the recombinant proteins was assessed by SDS-PAGE, while the molecular weights were measured by MALDI-TOFMS (Supplementary Figure 3). Purified recombinant proteins were incubated with proteins extracted from BMEC immobilized in microtiter wells in ELISA. The results confirmed binding of all five recombinant ligands to the BMEC proteins (Fig. 3A). Of note, control experiment without recombinant proteins (negative control, also used for background subtraction) confirmed the specificity of the assay. Two proteins, PilW^16^ of *Francisella* known to not interact with BMECs and OspA^17^ of neuroinvasive *Borrelia* known to adhere on BMEC were also included in assay. No interaction between PilW and BMEC lysate was observed (mean RFU 133.3, std. error of mean 40.96), while the binding of OspA to the proteins of BMEC was strong (mean RFU 6505, std. error of mean 647.8). Among five pneumococcal ligands binding affinity of adhesion lipoprotein (Spr0906) was the strongest (mean RFU 6363, std. error of mean 307.2) (Fig. 3A).

**Table 2:** Potential pneumococcal ligands selected for ligand-BMEC interaction.

No.	Entry (UNIPROT)	Protein name	Locus	Amino acids encompassed in recombinant form	Mr (KDa)	
					Theoretical	Observed in SDS-PAGE
1	Q8DQ09	Adhesion lipoprotein	spr0906	G56 – K294	55.183	≈55
2	Q8CYB8	Hypothetical protein	spr1730	G21 – E219	50.481	≈50
3	Q8CZ52	LPXTG-motif cell wall anchor protein	spr0440	A276 – A1021	111.461	≈111
4	Q8DQ98	Hypothetical protein	spr0777	G88 – G159	36.367	≈36
5	Q8DPQ2	Pneumococcal histidine triad protein A	spr1061	G35 – S823	118.780	≈118

We performed immunocytochemistry to corroborate interaction between pneumococcal ligands and BMECs, in which recombinant ligands were incubated with the BMECs cultured on collagen coated coverslips. Bound ligands were visualized with anti-6x His antibody conjugated with FITC. Binding of Spr0906 and Spr0777 on endothelial cells was evidently stronger than other three pneumococcal ligands (Fig. 3B). Binding for PilW and OspA on endothelial cells were in correlation with affinities observed in ELISA (Fig. 3A,B).

### Antigenicity of the selected ligands

Five potential ligands of *S*. *pneumoniae* selected for recombinant production were analyzed *in silico* to evaluate their antigenicity and their capacity of triggering humoral immune response. Three out five proteins (Spr0906, Spr0440 and Spr1061) were predicted as antigenic proteins (probability > 0.8) and 2 out of 5 proteins (Spr0906 and Spr0440) were predicted as potential vaccine candidates (See supplementary Table 4), from which Spr0440 has been used already in vaccine design (See supplementary Table 1). Adhesion lipoprotein was predicted to have one T-cell epitope and conserved in 6 pathogenic strains of *S*. *pneumoniae* (See supplementary Table 4).

### Juxtaposition of surfome interacting with human BMECs and pansurfome of *S*. *pneumoniae* R6

When we compared surface-exposed proteins putatively binding to human BMECs with pansurfome of *S*. *pneumoniae* R6, we found that 3 out of 11 cell-wall anchored proteins predicted from the R6 genome were identified in our study. Similarly, two out of 32 proteins possessing SP1-type signal peptide, 2 out of 39 lipid-anchored proteins, 9 out of 107 with one transmembrane domain and 6 out of 368 multi-transmembrane domain proteins were observed in our study (Supplementary Table 2).

## Discussion

Interplay between cells and their surroundings is markedly mediated by proteins expressed on the cell surface. Meningitis-causing *S*. *pneumoniae* displays a wide arsenal of molecules associated with pathological processes^27^. However, their contribution in pneumococcal meningitis is not fully unfolded and the mechanisms by which pneumococci cross the BBB are not well understood^1,4^. Available data suggest that pneumococcus traverses the BBB transcellularly and few well-known molecules participate in this process activating specific cell surface receptors on the NVU^3,28^. Detailed knowledge of the ligand-receptor interaction and downstream cell activation events, could increase the chances to effectively intervene the BBB translocation and succeed in the development of strategies for disease cure and prevention^29^.

In the present study, we have performed a proteomics-based experiment followed by an integrative analysis with combination of bioinformatics tools and comprehensive literature review for the identification and selection of proteins probably interacting with human BMEC. The strategy proposed in this study requires a proper labeling of the proteome of *S*. *pneumoniae* with biotin. Biotin labeling has been successfully applied to study protein-protein interactions or protein characterization of several biological models^30–32^. The biotinylation of the surface proteome of the intact pneumococcus cells was attempted by us, however with little success (data not presented). This might be because of the thick capsule present on pneumococci. Thus, the alternative approach, biotinylation of the whole protein extract was used here. We succeeded in the labeling of most of the proteins species (as observed in Fig. 1A), which was crucial in the recovery of BMEC binding ligands and their identification with SWATH-MS. Gel-free proteomics has been successfully applied in the discovery of new antigenic proteins exposed on the surface of several pathogenic bacteria^30,33,34^. In addition, the data independent acquisition method (SWATH-MS) has proven to be a highly sensitive and robust technology reducing signal variation and considerably increasing number of accurate peptides^35,36^.

In this work, we identified in a fast and reliable way tens of proteins that plausibly bind human BMEC. As the surface-exposed proteins play a critical role in the interaction with the extracellular milieu^36,37^, in the downstream analysis we focused only on cell wall associated proteins. To do so, we proposed a detailed bioinformatic workflow for the systematic analysis of MS derived dataset to categorize the proteins according to subcellular location and obtain relevant biological information. At first, after subcellular prediction with bioinformatic tools, the list of identified candidates was divided in to the surface-exposed and cytoplasmic proteins. Cytoplasmic proteins observed in the present study might be due to non-specific interaction with either the surface proteome of BMEC or with the surface-associated proteins of pneumococci. Nevertheless, we do not exclude the possibility that some proteins could participate in pathological processes (e.g. SP0966 and enolase have been associated with adherence or pathogenesis, respectively^38,39^).

Among shortlisted 22 exclusively surface-exposed proteins, we observed presence of 15 membrane proteins, which proves the suitability of the experimental approach used in this study. The membrane-attached proteins possess high or moderate hydrophobic domains along the peptide sequence^31,40^ and are difficult to resolve applying gel based proteomic platforms such as 2D-PAGE. Moreover, in the list of potential candidates around 45% corresponded to proteins with unpredicted functions in GO, which makes them highly interesting for further studies on molecular characterization, participation in virulence and pathogenesis, or assess their role in the protein-protein interactions. The GO annotation search for the rest of 55% protein candidates revealed biological processes such as transmembrane transport, biosynthetic mechanisms or cell adhesion. The later are of the main interest in the present study as they could potentially interact with the cells of neurovascular unit. Another finding in this study was identification of proteins participating in the transmembrane transport or ion binding such as the manganese ABC transporter (PsaA) and zinc metalloprotease (ZmpB). It has been reported previously that during the infection host cells recruit divalent cations (Fe^2+^, Zn^2+^, or Mn^2+^) as a natural mechanisms to combat bacteria^41–43^. In response, many pathogens use sensing system to detect ionic changes and recruit essential ions to survive^44^. Thus, the identification of proteins related to metal transport might be related to this process.

The suitability of the proteomics-based screening proposed in this study was evaluated by literature review. Ligands such as CbpA and Ply, identified in our study, were also described previously as interacting proteins with human BMEC^6,45^. Similarly, role of PspA in meningitis is also described previously^46^, which supports the experimental approach proposed in this study.

It is noteworthy that the protein Spr0075, shortlisted as one of the potential BMEC interacting candidate, showed homology with protein SP0082 (cell wall anchor protein, *S*. *pneumoniae* TIGR4) that contains fibronectin-binding motifs important in binding to extracellular matrix proteins^5,47^. Interestingly, proteins such as PhtA, MalX, IgA1 contribute in the development of pneumonia or sepsis^12,48,49^, meanwhile, PsaA and PfbA participate in adhesion of epithelial cells^50,51^. Therefore, it is tempting to speculate that these proteins may also possess a role in the adhesion of pneumococcus to human BMEC, which is a prerequisite for transient translocation of pathogen across BBB^52^.

The protein interactive analysis performed as the last step of the bioinformatic analysis revealed potential associations among the set of identified proteins. The STRING showed two main networks with overlapping five proteins candidates (Ply, IgA1, ZmpB, PfbA and PspC). Out of the five candidates,Ply, PspC and ZmpB are already reported to associate with meningitis^10,53^.

Based on the data retrieved after bioinformatic analyses and literature review, five proteins were selected for further validation. Our selection was carefully decided to study proteins for which few information is available or whose role in meningitis is not clear or has not been investigated. Binding of these ligands to human BMEC was confirmed with ELISA and immunocytochemistry, while binding affinity of Spr0906 was apparently high. Spr0960was also observed as antigenic and immunogenic protein with bioinformatics analysis. Unfortunately, we were not able to confirm interaction between above said proteins and BMECs with the help of genetic tools e.g. using knockout *S*. *pneumoniae* mutants or overexpression of these proteins in a non-pathogenic bacteria such as *Lactobacillus lactis* and assay for gain-of-function. Permits for such experiments are not currently available at our institution due to restriction on work with genetically modified organisms.

The BBB selectively facilitates the transport of nutrients and restricts passage of pathogenic bacteria. Considering the complexity of biological processes triggered by *S*. *pneumoniae* on the BBB it is possible to assume that tens (even hundreds) of proteins of this pathogen can interact simultaneously with different receptors expressed on the cells of neurovascular unit activating multiple signal cascades. Thus, this approach provides main interactors of pneumococcus with BMEC as it was also confirmed in the literature review. However, role of the most of interactors in meningitis has not been described.

Overall findings show that the high-throughput proteomics coupled with systematic bioinformatics pipeline could be one of the optimal approaches in the simultaneous identification of proteins candidates with high probability to interact with host cells and filter them as per their subcellular localization. Although the pipeline was used to identify proteins of *S*. *pneumoniae* interacting with human BMEC, it is possible to implicate this workflow to other pathogens and host cell types to identify and characterize cellular receptors and their ligands.

Given that the datasets generated from MS analysis could include false positives identifications which are normally excluded using an internal decoy database, pipelines for the identification of interacting proteins should also integrate filters (e.g. various bioinformatics analyses) for the selective exclusion of false positive interactors or non-relevant proteins. It is important to note that, several combinations of the bioinformatic tools can be applied for selection of the most probable interactors. The bioinformatics pipeline may vary depending on pathogen or the host cells under study, moreover selection of proper bioinformatics tools also needs rigorous literature review as well as robust consensus of the generated data from bioinformatics. We would like to highlight that high-throughput approaches and sensitive analytic tools (like LC-MS) may generate false positives hits. Protein might be present in the list obtained from LC-MS analysis as a potential interactor with the host cells, but after all it might not be important in the infection process and/or as vaccine targets. Detailed *in-silico* analysis followed by wet lab experiments (*in-vitro* experiments on cell cultures, genetic manipulation of pathogen, *in-vivo* experiments etc.) are necessary to rule out false positive hits.

In summary, several combinations of the bioinformatic tools can be applied to screen interacting protein candidates. We succeed in the identification of interacting proteins through one of those possible combinations (i.e. the proposed workflow) and we revealed at least five proteins of pneumococcus that may interact with human BMECs. Further research is still needed to corroborate interaction of identified proteins of pneumococcus with BMECs. It is necessary to dissect their interaction network and obtain data to find out importance in the infection process for example using genetic tools like knockout gene mutants.

## Methods

### Bacterial culture

*S*. *pneumoniae* (clinical isolate SPH) was plated on Columbia agar blood base containing 5% (v/v) sheep blood and single isolated colony was grown in 100 mL of Todd Hewitt Broth at 37 °C and 5% CO_2_ until OD_600_ = 0.4 (mid-exponential phase). The neuroinvasive strain of *S*. *pneumoniae* used in this study was isolated from the cerebrospinal fluid of a meningitis-suffering patient hospitalized in Louis Pasteur Hospital, Kosice, Slovakia. The isolate was characterized by phenotyping (biochemical tests) and genotyping (sequencing of lytA and rpoB genes) in the hospital laboratory.

### Human brain microvascular endothelial cells culture

Human BMEC (hCMEC/D3 cell line) was obtained from Merck/Millipore (Prague, Czech Republic). Cells were cultured in 25-mL cell culture flask coated with collagen type I (Sigma, USA) in EBM-2 medium (Lonza, UK) containing 10% FBS, gentamycin, 1.4 μM hydrocortisone (Sigma), 5 μg/mL ascorbic acid, 10 mM HEPES and 1 ng/mL bFGF (Sigma). Cells were incubated at 37 °C in a humid atmosphere of 5% CO_2_ until confluence. Cells were either harvested for protein extraction or incubated with proteins of pneumococci.

### Protein extraction of human BMEC

Confluent monolayer of human BMECs was gently scraped with 1 mL lysis solution (20 mM CHAPS, 300 mM NaCl, 0.1% sodium azide and 1x proteases inhibitors). Cells were disrupted with 5 cycles of sonication on ice (100% amplitude, 30 s). Proteins were recovered by centrifugation (10,000 × g, 5 minutes, 4 °C), quantified with Bradford method, aliquoted and stored at −80 °C until use for validation of ligand-receptor interactions.

### Protein extraction of pneumococci and biotinylation

100 mL of culture were centrifuged at 5,000 × g for 10 minutes at 4 °C and pelleted bacteria were washed two times with sterile PBS. Cells were resuspended in sterile water supplemented with 1x proteases inhibitors (Sigma). 5 cycles of heat-shock (−80 °C and room temperature) were carried out and supernatants were removed after centrifugation at 26,000 × g for 10 minutes (this step removes water soluble proteins, mainly cytoplasmic, and enriches hydrophobic candidates e.g. membrane proteins). Afterwards, pellets were resuspended in non-denaturating lysis solution containing 20 mM CHAPS, 300 mM NaCl, 0.1% sodium azide and 1x proteases inhibitors. Samples were sonicated 15 cycles on ice (100% amplitude, 30 s). Subsequently, proteins in supernatant were collected by centrifugation at 26,000 × g for 10 minutes and biotinylated with EZ-Link Sulfo-NHS-LC-Biotin (Thermo Fisher Scientific) in ratio 5:1 (5 mg of protein/mg of biotin), according to manufacturer’s instructions. Biotinylation was confirmed with NeutrAvidin (Thermo Fisher Scientific) capture followed by SDS-PAGE as per manufacturer’s instructions. Protein samples were quantified with Bradford method, aliquoted and stored at −80 °C until incubation with human BMEC.

### Ligand-cell interaction

To carry out the ligand-cell interaction, 200 μg of biotinylated proteins of pneumococci were incubated with confluent monolayer of human BMECs grown in 25-mL cell culture flask for 1 hour at 37 °C in presence of 5% CO_2_. Unbound proteins were removed by two washes with Dulbecco’s-PBS (Sigma). Human BMEC with bound proteins of pneumococci were scraped in 2 mL of PBS. Cells were harvested by centrifugation (3,000 × g, 10 minutes). Supernatant was saved and kept on ice until use (S1). Pelleted cells were resuspended in 200 μL of lysis solution (cell surface protein isolation kit, Thermo Fisher Scientific) and incubated 30 minutes on ice (during incubation cells were vortexed for 5 seconds after every 5 minutes). Finally, the supernatant S1 was added and cells were disrupted with 5 cycles of sonication on ice (100% amplitude, 30 s). Debris was removed by centrifugation (10,000 × g, 5 minutes, 4 °C) and protein fraction (supernatant, S2) was kept on ice to proceed immediately with capturing of biotinylated proteins as described below.

For negative control biotinylated proteins were incubated in collagen coated flask (without BMECs) as described above. After two washes with Dulbecco’s-PBS any protein attached to collagen was scrapped in 2 mL PBS, centrifuged (10,000 × g, 5 minutes, 4 °C) and supernatant was used for capturing of biotinylated proteins as described below.

In parallel, dot blot was carried out to confirm the presence of biotinylated proteins in the protein extract. Biotinylated proteins of pneumococci and protein extract of human BMEC were used as input and negative control, respectively. Details are shown in supplementary method 1.

### Capture of biotinylated proteins from cell extract and protein identification

Biotinylated proteins were recovered from the cell lysate (SN2) with NeutrAvidin agarose beads (Thermo Fisher Scientific) according to manufacturer’s instructions and were identified by LC-MS SWATH analysis. Detailed steps of the protein capture and the complete protocol of the MS analysis are shown as supplementary method 2 and 3, respectively.

### A workflow of bioinformatic analysis: prediction of subcellular localization, ontology and protein interactions

**Figure 4:**
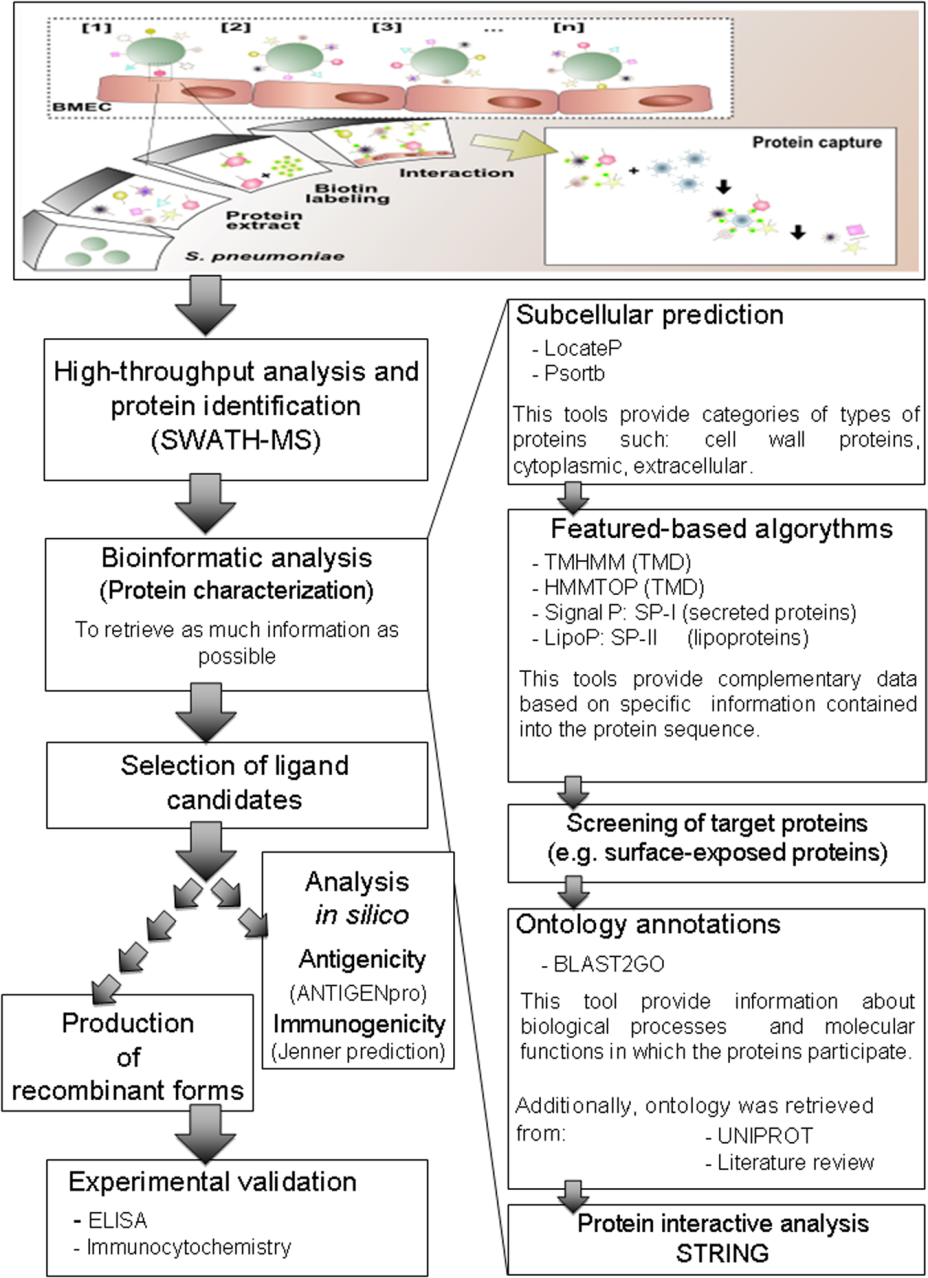
Overview of experimental and bioinformatic pipeline proposed in the study. Proteome of pneumococci was biotinylated and incubated with human brain microvascular cells (BMEC). Multiple interactions among various labeled proteins and surface of BMEC are represented as [1, 2, 3… n]. Potentially interacting ligands recovered with NeutrAvidin capture beads were identified by SWATH-MS. A systematic bioinformatic workflow was established for the proper selection of candidates for recombinant production and further validation of the proposed scheme. First, proteins were categorized based on the subcellular location, which followed by application of featured/based algorithms to retrieve detailed information like presence of transmembrane domains, prediction of signal peptide etc. Third, selection of proteins with high probability to bind host cells based on surface exposure. Fourth, characterization of candidates based on the ontology analysis, data search in protein repositories and literature. As a last bioinformatics analysis step, prediction of interactive proteins was performed to assess role of proteins in the process of pathogenesis. Finally, ligand candidates were selected for recombinant production and the proposed workflow was validated with ELISA. Selected ligands were evaluated *in silico* for antigenicity and immunogenicity. The proposed workflow was validated with ELISA and immunocytochemistry using recombinant form of ligands.

To shortlist the protein candidates with high probability to interact with BMECs, a three-step bioinformatics analysis was applied (Please see brief description of the tools in supplementary method # 5 in supplementary information). The first, segregation of protein candidates based on subcellular localization, the second, ontology analysis and finally the prediction of protein interactions (Fig. 4). Primary predictions of subcellular localization were assigned by using the web-based algorithm LocateP (http://www.cmbi.ru.nl/locatep-db/cgi-bin/locatepdb.py)^7^ and Psortb (http://www.psort.org/psortb/)^54^. These algorithms group the protein candidates in more general categories (e.g. cytoplasmic, membrane proteins or cell wall proteins). The segregated candidates were further categorized based on: 1. potential of transmembrane helices, 2. type-I signal peptides and 3. potential to have lipid moiety, using featured-based algorithms. TMHMM 2.0 (http://www.cbs.dtu.dk/services/TMHMM-2.0)^55^ and HMMTOP (http://www.enzim.hu/hmmtop/) were used for prediction of transmembrane helices^56^. SignalP 3.0 (http://www.cbs.dtu.dk/services/SignalP)^57^ was applied for prediction of type-I signal peptides (those proteins containing only a cleavable type-I signal peptide as featured sequence were classed as secreted). Whereas, LipoP (http://www.cbs.dtu.dk/services/LipoP)^58^ was used for identification of type-II signal peptides (characteristic of lipoproteins). Subcellular predictions and feature-based algorithms segregate a list of surface-exposed proteins.

For those proteins that showed potential of surface exposure, Blast2GO (https://www.blast2go.com) was performed to retrieve annotations from the characterized proteins, while in case of non-annotated proteins, annotations were retrieved from their orthologs (>95% identity). When Blast2GO failed to provide annotation for some protein candidates (either from characterized protein or from orthologs), data search was performed in UniProt Knowledge database (http://www.uniprot.org/) and literature to compile relevant data.

In the final step of bioinformatic workflow, an interactive analysis was carried out for surface-exposed proteins based on the Search Tool for Retrieval of Interacting Genes (STRING, https://string-db.org) to evaluate possible associations of proteins of interest in pathogenesis^59^.

### Prediction of antigenicity and immunogenicity

Antigenicity and immunogenicity of protein candidates was evaluated using web free predictors. ANTIGENpro algorithm (https://scratch.proteomics.ics.uci.edu) (a sequence-based alignment pathogen-independent predictor of the whole protein sequence) was used for prediction of antigenicity. Proteins larger than 1000 size were analyzed in chains. Protein immunogenicity was evaluated with Jenner Predict algorithm (http://14.139.240.55/vaccine/validation.html) for prediction of protein vaccine candidates.

### Description of total number of surface-exposed proteins

In order to assess a total number of surface-exposed proteins present in reference strain *S*. *pneumoniae* R6, a repository from LocateP (http://www.cmbi.ru.nl/locatep-db/cgi-bin/locatepdb.py) was retrieved. Shortlisted surface-exposed candidates from our study were compared to surface-exposed proteins predicted in R6.

### Synthesis of recombinant form of the shortlisted proteins

Based on the bioinformatic analyses, protein candidates were shortlisted and their recombinant forms were produced (See Supplementary Dataset 2 for further details). In short, the gene fragments encoding proteins were amplified by PCR from genomic DNA of *S*. *pneumoniae*. List of the designed primers, overhangs of restriction sites used for downstream cloning and length of amplicons are shown in Table 3. Amplified fragments were digested with restriction enzymes (Thermo Fisher Scientific, Slovakia) as shown in Table 3, as per the manufacturer’s instruction and ligated into pQE-30-mCherry-GFP plasmid (in-house modified vector pQE-30 UA, Qiagen, Supplementary Figure 4). Selection and culture of clones are detailed in supplementary method 4.

**Table 3:** Primers used to produce recombinant forms of the ligands.

No.	Protein/(Gene)	Sequence used to design primers	Primer	Sequence (5′-3′)	Amplicon length (bp)
1	Adhesion lipoprotein(*Spr0906*)	AE007317.1*: nt891426 to nt892361	*spr0906*- sense	TATAGATCTGGTGACTTGAATGATGTTCGG	717
			*spr0906*- antisense	TTTGTCGACGGTCTTGTCATTTTGTGGGTC	
2	Hypothetical protein(*spr1730*)	AE007317.1*: ntc1708366 to nt1707692	*spr1730*- sense	TATGGATCCGGGAATTTTGGAATTCCTTCT	597
			*spr1730*- antisense	TTTGTCGACTTCCAACTTCCCAAAAGCCAC	
3	LPXTG-motif cell wall anchor protein(*spr0440*)	AE007317.1*: nt441113 to nt446092	*spr0440*- sense	TATGGATCCGCAGGTCACCGTAACGGGGTT	2238
			*spr0440*- antisense	TTTGTCGACTGCTAGGTCTCCTCCAACTTCGCT	
4	Hypothetical protein (*spr0777*)	AE007317.1*: nt771852 to nt772961	*spr0777*- sense	TATGGATCCGGTGTTGTCTATCTCTTACCTATTTTG	216
			*spr0777*- antisense	TTTGTCGACCCCCACACTATTTGATACGCT	
5	Pneumococcal histidine triad protein A (*Spr1061*)	AE007317.1*: ntc1059416 to nt1056930	*spr1061*- sense	TATGGATCCGAGTTGGGACTGTATCAAGCT	2367
			*spr1061*- antisense	TTTGTCGACACTTACAGATGAAGGATTACTTCC	

*Shows Genbank accession number followed by nucleotide positions spanning the gene. Restriction sites are depicted with underlined nucleotides. AGATCT – *Bgl*II, GGATCC – *BamH*I and GTCGAC – *Sal*I.

Presence of encoding gene in transformants was confirmed by sequencing (vector specific primers UA Insertom F and R, presented in supplementary Table 3). Protein expression was induced with 1 mM IPTG (Fermentas, Slovakia) at 20 °C. Expression level was monitored under the fluorescent microscope (all proteins are GFP tagged). After induction, cells were pelleted (17,880 × g for 10 minutes) and lysed in lysis buffer (0.03 M Na_2_HPO_4_, 0.5 M NaCl, 0.001% Tween 20, 10% glycerol) with four freeze-thaw cycles followed by sonication on ice (2 cycles; 30-s pulses, 100% amplitude). Proteins were purified with nickel affinity chromatography (Ni-NTA agarose beads, ABT, Spain) as per manufacturer’s instructions. Eluted fractions were evaluated by SDS-PAGE and MALDI-TOF MS. Protein concentration was measured and aliquots of purified proteins were stored at −20 °C in 20% glycerol until use.

### Confirmation of binding of ligands of pneumococci with the proteins of human BMECs

Protein-protein interaction was assessed using a direct ELISA. Microtiter plates were coated with 150 μg/mL of total protein extract of human BMEC in coating buffer (PBS, pH 7.2) overnight at 4 °C. Plates were washed three times with PBS containing 0.1% Tween-20 and then were incubated with 100 μL (100 μg/mL) of each recombinant protein for 1 hour at room temperature and washed three times with PBS containing 0.1% Tween-20. Proteins of pneumococcus bound to human BMEC were detected by incubation with anti-GFP antibody conjugated to HRP diluted in PBS containing 0.1% Tween-20(1:20,000, Biotium) for 1 hour at room temperature and washed again three times. Plates were incubated with HRP substrate solution and later stopping solution was added (LI-COR, Biosciences). Finally, the signals were measured at 700 nm (Odyssey CLx, LI-COR bioscience). As an input control, 10 μg of each recombinant protein were coated on the wells and detected with HRP conjugated anti-GFP antibody. As a negative control 15 μg of cell extract of human BMEC were coated on the wells and incubated with HRP conjugated anti-GFP antibody. PilW protein of *Francisella* (known to not interact with BMECs) and OspA of neuroinvasive *Borrelia* (known to interact with BMECs) were also included in the experiment as known negative and positive controls. Both proteins were produced in our laboratory previously to assess their binding affinity to BMECs^16,17^. ELISA was performed three times and in every experiment each recombinant protein was in triplicate.

### Confirmation of binding of ligands of pneumococci to the cultured BMECs

In brief, BMECs were cultured on the coverslips coated with collagen type I (Sigma, USA) in 6 well culture plates in supplemented EBM-2 medium (Lonza, UK) as described above until 70% confluency. Cells were washed two times with non-supplemented EBM-2 medium to remove any dead cells. Cell were incubated with purified recombinant ligands (25 μg resuspended in 2 mL EBM-2 medium) for 2 hours at 37 °C in 5% CO_2_. Cells were washed three times with PBS, fixed with 4% paraformaldehyde for 10 min and washed again three times with PBS containing Tween 20 (0.05%, PBST). Cells were then incubated with anti-His antibody conjugated with FITC (diluted in PBS with 1% BSA - blocking buffer, 1:400; Abcam, UK) for 2 hours in dark at room temperature with gentle shaking. After 3 washings with PBST cells were incubated in DAPI (final concentration 1 μg/ml in PBS; AppliChem, Switzerland) for 30 minutes. Cells were rinsed once with PBS, coverslips were taken out of the wells, dipped in ultra-pure ethanol for 2–3 seconds and mounted with mounting medium (Fluoroshield, Sigma). In case of negative control recombinant ligands were excluded. PilW protein of *Francisella* (known to not interact with BMECs) and OspA of neuroinvasive *Borrelia* (known to interact with BMECs) were also included in the experiment as known negative and positive controls. Photo documentation was performed on LSM-710 microscope (Zeiss, Germany) using 359–461 nm filter for DAPI and 495–519 nm filter for FITC. The assay was performed in biological triplicates.

### Data availability statement

The datasets generated during and/or analysed during the current study are available from the corresponding author on reasonable request.

## Electronic supplementary material

Supplementary information

Supplementary Dataset 1

Supplementary Dataset 2
