# Supplementary material for: Proteomic and bioinformatic pipeline to screen the ligands of S. pneumoniae interacting with human brain microvascular endothelial cells
Source: Sci Rep. 2018 Mar 27;8:5231. doi: 10.1038/s41598-018-23485-1 (PMC5869694; doi:10.1038/s41598-018-23485-1)
Supplement: Supplementary file 1 — Supplementary information [file 41598_2018_23485_MOESM1_ESM.doc]

**Proteomic and bioinformatic pipeline to screen the ligands of *S. pneumoniae* interacting with human brain microvascular endothelial cells**

**Irene Jiménez-Munguía^1^, Lucia Pulzova^1^, Evelina Kanova^1^, Zuzana Tomeckova^1^, Petra Majerova^2^, Katarina Bhide^1^, Lubos Comor^1^, Ivana Sirochmanova^1^, Andrej Kovac^2^ & Mangesh Bhide^1,2^*.**

**SUPPLEMENTARY FIGURES**

**
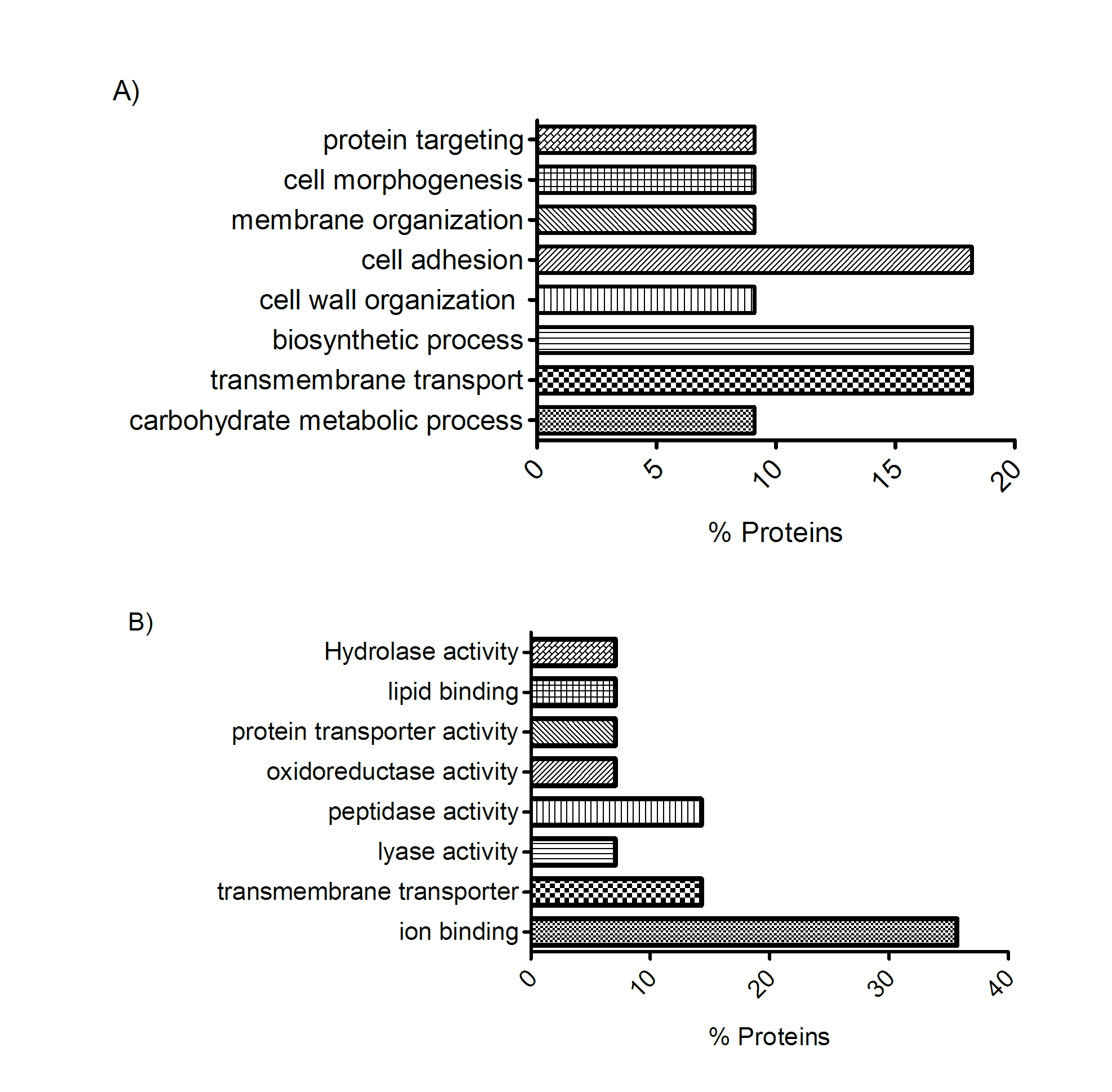
**

**Supplementary figure 1. Ontology analysis of ligands of pneumococci putatively interacting with human BMEC in our study.** GO analysis was performed with Blast2GO, A) Gen ontology terms identified for the biological processes. B) Gene ontology terms identified for the molecular function category. % proteins represent protein species involved in each specific category.

**
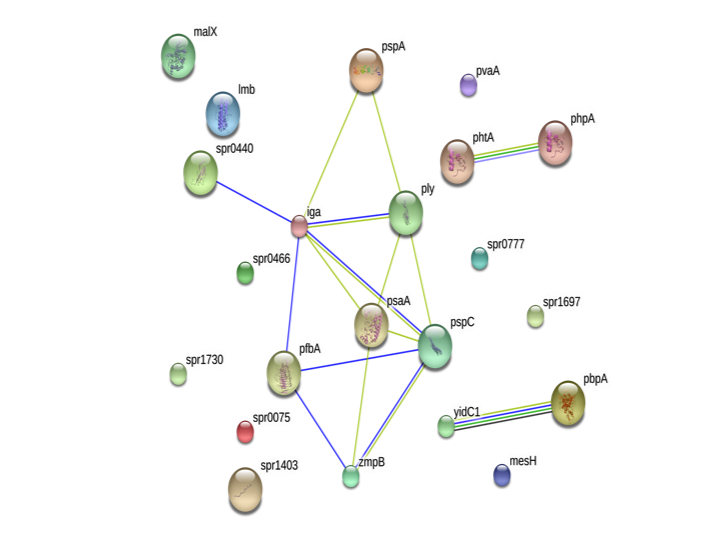
Supplementary figure 2. Protein interactive analysis predicted by the Tool for Retrieval of Interacting Genes (STRING).** Interactive proteins are connected by colored lines. Proteins connected with a faint green line form network 1 and proteins linked with a blue line make network 2. Lines interconnect colored nodes, big nodes describe known proteins and small nodes represent proteins with less information. *, proteins described in literature associated with meningitis.

**
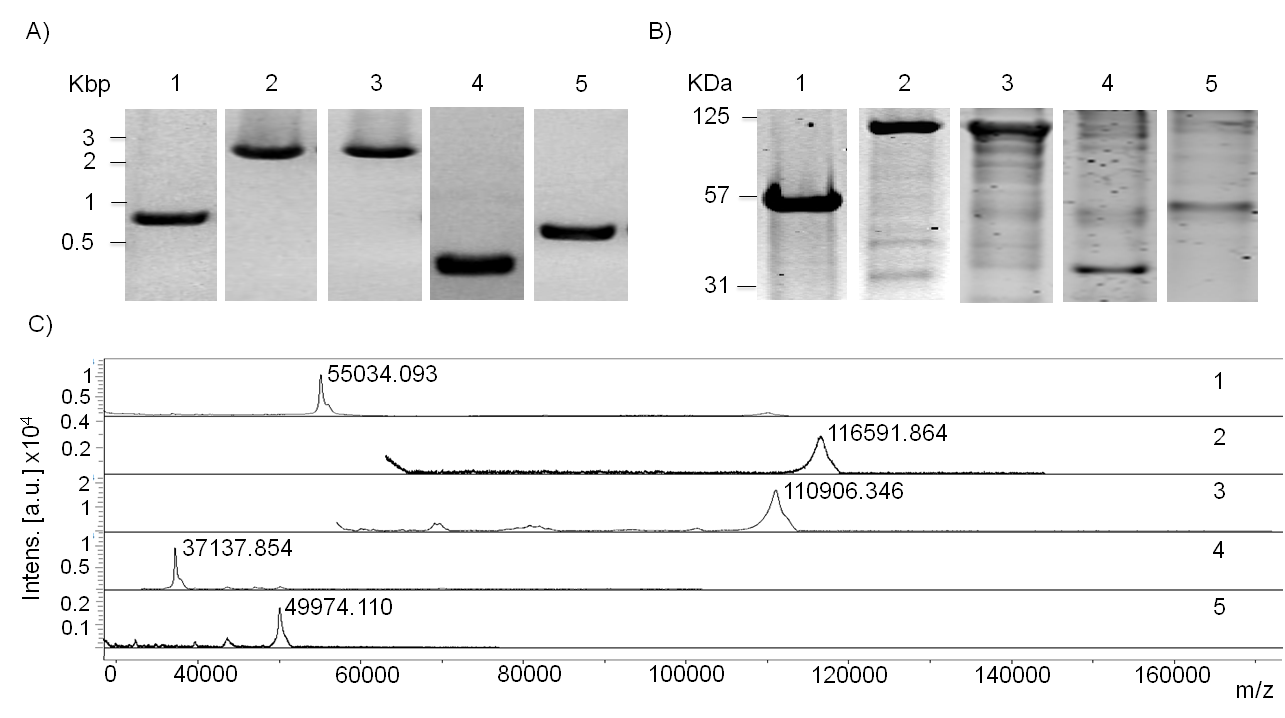
Supplementary figure 3. Production of recombinant forms of the selected protein candidates.** Panel A, amplicons of the five gene coding fragments of potential ligands of pneumococci resolved on agarose gel; panel B, purified recombinant proteins separated with SDS-PAGE; panel C, molecular mass of recombinant proteins confirmed with MALDI-TOF/MS. Lane 1, adhesion lipoprotein (Spr0906); Lane 2, pneumococcal histidine triad protein A (Spr1061); Lane 3, endo-β-N-acetylglucosaminidase (Spr0440); Lane 4, unknown protein (Spr0777); and Lane 5, unknown protein (Spr1730).

**
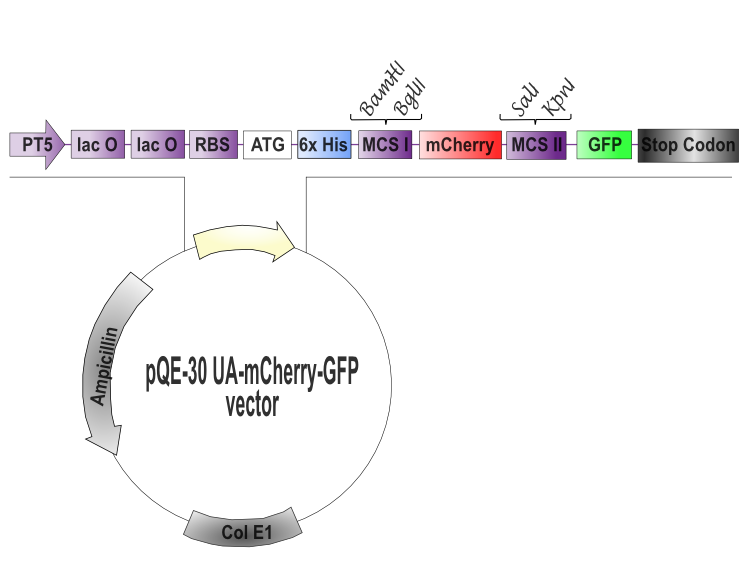
Supplementary figure 4. Vector map of pQE-30-mCherry-GFP plasmid (4880 bp).** Col E1 origin of replication, ampicillin resistance gene, *PT5* T5 promoter, *lac O* lac operator, RBS ribosome binding site, ATG Start codon, *6x*His tag sequence, MCSI/MCSII multiple cloning sites, mCherry- red fluorescent protein that serve as stuffer, GFP-green fluorescent protein and Stop codon.

**
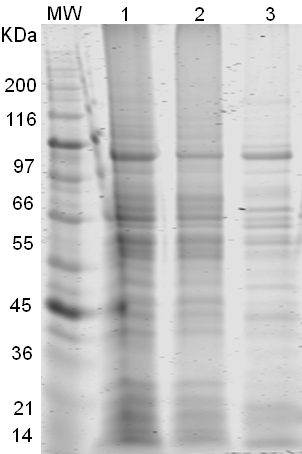
**

**Supplementary figure 5. Original figure for making the Figure 1A.** Lane 1, protein extract of pneumococci prior to biotinylation separated on SDS-PAGE. Lane 2, biotinylated proteins were incubated on NeutrAvidin capture beads, eluted with 50 mM DTT and separated on SDS-PAGE. Lane 3, flow through recovered after incubation of biotinylated proteins with NeutrAvidin beads. Please note that presence of proteins in the flow through might be due to saturation (overload of the proteins) of NeutrAvidin capture beads or because of insufficient labeling of particular protein species or combination of both.

| **A)**  **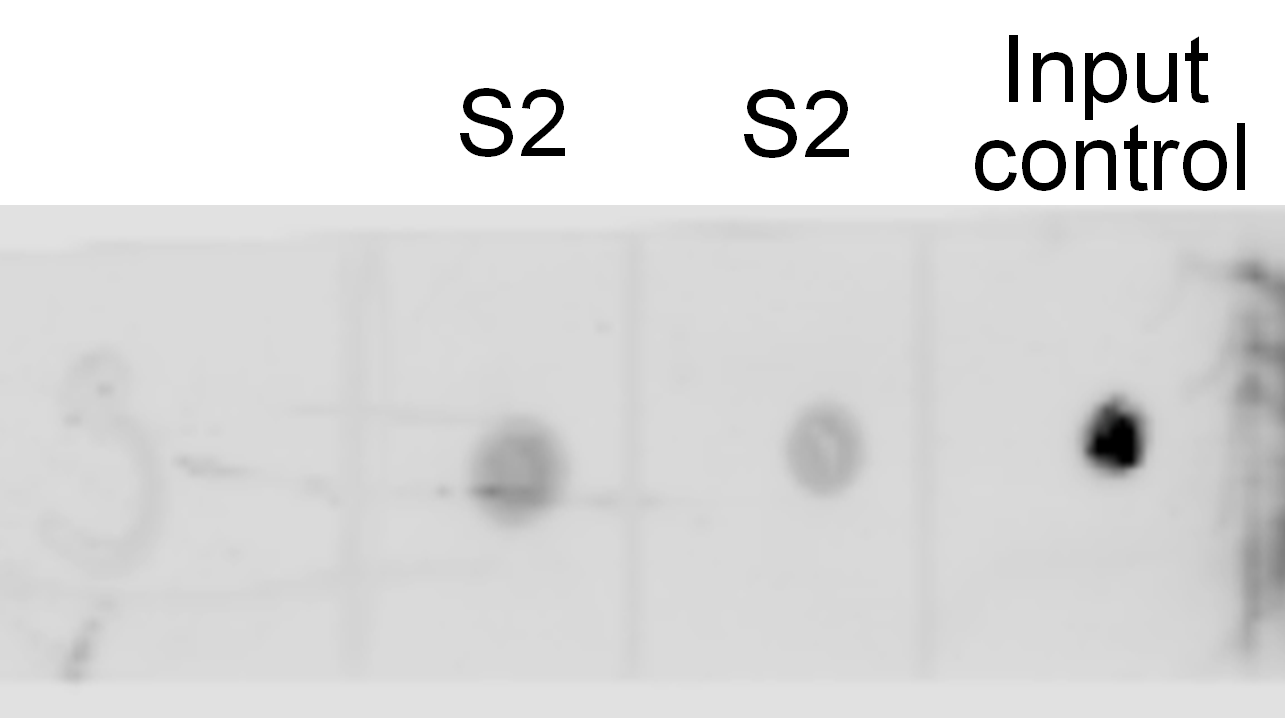** | **B)**  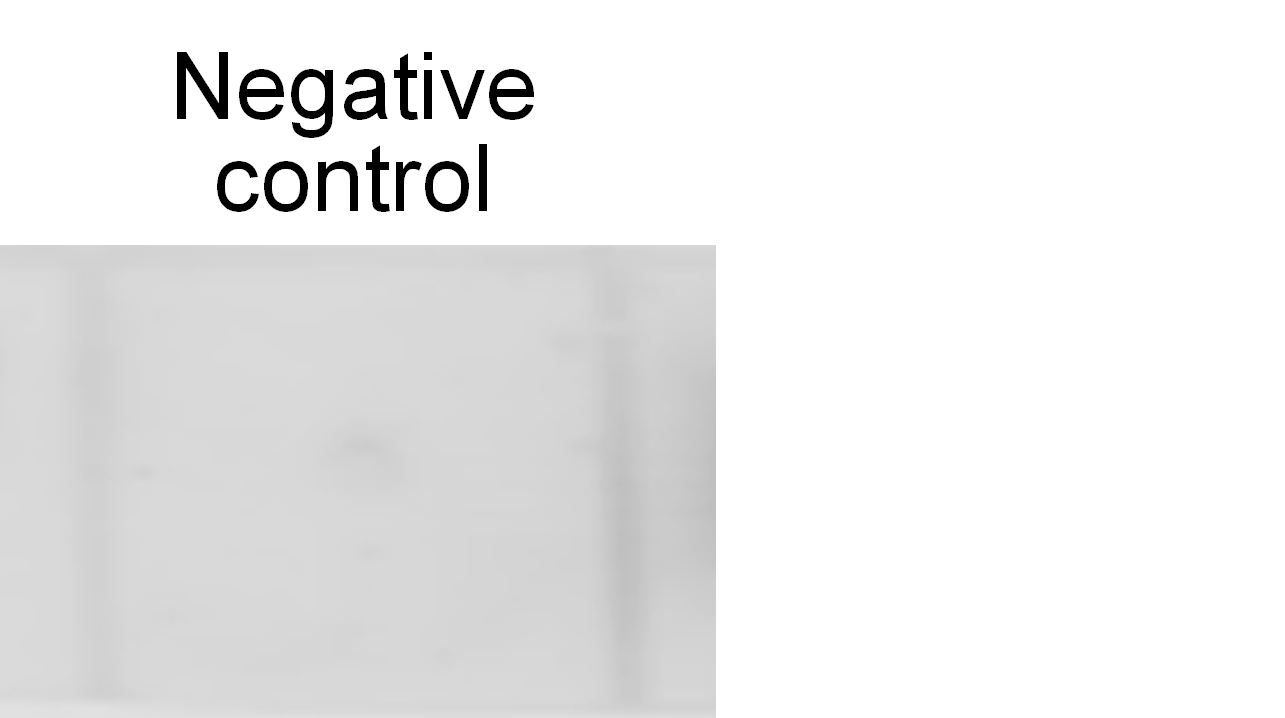 |
| --- | --- |

**Supplementary figure 6. Original figures for making the Figure 1B.** A) Protein extract of BMEC obtained after incubation of biotinylated proteins of pneumococci with BMEC was spotted on the membrane and detected with IRdye®800 Streptavidin (S2) in dot blot in duplicate. Biotinylated proteins of pneumococci were spotted on membrane and detected with IRdye®800 Streptavidin (input control). B) Total protein extract of human BMECs was spotted on membrane and incubated with IRdye®800 Streptavidin (negative control).

**SUPPLEMENTARY TABLES**

| Supplementary table 1. Results of BLAST2GO, ontology analysis and literature review of identified surface proteins | | | | | | | | | | | | | | | |
| --- | --- | --- | --- | --- | --- | --- | --- | --- | --- | --- | --- | --- | --- | --- | --- |
| Entry | Protein name | Locus | Ontology^a^ | | | | | Literature^b^ | | | | | | | |
|  |  |  | Transport | Ion binding | Adhesion | Peptidase | Other | VD | SD | RT | Meningitis | Sepsis | Adhesion | Other | References |
| P67294 | UPF0154 protein spr1697 | spr1697 | X |  |  |  |  | - | - | - | - | - | - | - |  |
| P59214 | Maltose/maltodextrin-binding protein | spr1918 | X |  |  |  |  | X | X | X |  |  |  |  | 1–3 |
| Q8DQW4 | Uncharacterized protein | spr0466 | X |  |  |  |  | - | - | - | - | - | - | - |  |
| Q8DQ02 | MesH protein | spr0916 | X |  |  |  |  | - | - | - | - | - | - | - |  |
| P0A4G3 | Manganese ABC transporter substrate-binding lipoprotein (Pneumococcal surface adhesin A) | spr1494 | X | X | X |  |  |  | X |  |  |  |  |  | 1 |
| Q8DQ09 | Adhesion lipoprotein | spr0906 | X | X | X |  |  |  |  | X |  |  |  | X | 4,5 |
| Q8DNE1 | Membrane protein insertase YidC 1 (Foldase YidC 1) (Membrane integrase YidC 1) (Membrane protein YidC 1) | spr1790 | X |  |  |  | X | - | - | - | - | - | - | - |  |
| Q8DR59 | Penicillin-binding protein 1A (PBP-1A) (Exported protein 2) | spr0329 |  | X |  |  | X | X |  | X |  |  |  |  | 1 |
| Q59947 | Immunoglobulin A1 protease (IgA1 protease) (IgA-specific zinc metalloproteinase) | spr1042 |  | X |  | X |  |  | X |  | X |  |  |  | 1,6 |
| Q8DQN5 | Zinc metalloprotease ZmpB | spr0581 |  | X |  | X |  |  |  | X |  |  |  | X | 1,7,8 |
| Q8CZ52 | Uncharacterized protein | spr0440 |  |  | X* |  | X | X |  |  |  |  |  |  | 9,10 |
| Q8DRK2 | Uncharacterized protein | spr0075 |  |  | X* |  |  | X |  | X |  |  |  |  | 8,11 |
| Q7ZAK5 | Pneumolysin (Thiol-activated cytolysin) | spr1739 |  |  |  |  | X | X |  | X | X |  | X | X | 9,10,12,13 |
| Q8CYI8 | Uncharacterized protein | spr1403 | - | - | - | - | - |  | X |  |  |  |  |  | 1,14 |
| Q8DN05 | Choline binding protein A | spr1995 | - | - | - | - | - | X |  | X | X |  | X |  | 9,15–18 |
| Q8DPY9 | Pneumococcal vaccine antigen A | spr0930 | - | - | - | - | - | X |  |  |  |  |  |  | 10 |
| Q8CWR4 | Histidine Motif-Containing protein | spr1060 | - | - | - | - | - | X |  |  |  |  |  | X | 14,19 |
| Q8CYB8 | Hypothetical protein | spr1730 | - | - | - | - | - | - | - | - | - | - | - | - |  |
| Q8DQ98 | Hypothetical protein | spr0777 | - | - | - | - | - |  |  |  |  |  |  | X | 20 |
| Q8DPQ2 | Pneumococcal histidine triad protein A | spr1061 | - | - | - | - | - | X |  | X |  | X | X |  | 4,10,19 |
| Q8DRI0 | Surface protein pspA | spr0121 | - | - | - | - | - |  |  | X | X |  |  |  | 21–23 |
| Q8CYC9 | Plasmin and fibronectin-binding protein A | spr1652 | - | - | - | - | - |  |  | X |  |  | X |  | 17,24 |

^a^Categories of the functional analysis were established according to BLAST2GO predictions.

^b^ Categories assigned based on literature review; VD: vaccine design; SD: Serodiagnostics; RT: includes processes such as colonization of respiratory tract or pneumonia.

- Not assigned function or not found in literature.

* assigned by 99% identity in *S. pneumoniae* TIGR4

| Supplementary table 2. Summary of surface proteins identified in our study and their comparison with proteome of reference strain *S. pneumoniae* R6 | | | |
| --- | --- | --- | --- |
| Protein category | # identified proteins  in this study | # predicted proteins in  *S. pneumoniae* R6 genome | Identified/predicted (%) |
| Cell wall | 3 | 11 | 27 |
| Secretory | 2 | 32 | 6 |
| Lipoprotein | 2 | 39 | 5 |
| Membrane 1TMD | 9 | 107 | 8.4 |
| Membrane >1TMD | 6 | 368 | 1.6 |

Predicted proteins mean the total proteins contained in the *S. pneumoniae* R6 genome estimated *in silico* as surface-located. TMD means transmembrane domain. Categories were established according to LocateP.

| Supplementary table 3. Vector specific primers used to confirm presence of insert gene in transformants | |
| --- | --- |
| Primer | Sequence (5´- 3´) |
| UA Insertom F | CGCATCACCATCACCATCACG |
| UA Insertom R | ACCAAATTGGGACAACACCAGTG |

| Supplementary table 4. Protein antigenicity and immunogenicity evaluated on protein ligand candidates | | | | | | | | |
| --- | --- | --- | --- | --- | --- | --- | --- | --- |
| Entry UNIPROT | Protein name | Gene name | Protein chains | Predicted probability of antigenicity | Predicted as potential vaccine candidates | T-cell epitope  (number, amino acid sequence) | Conserved among pathogenic strains | Homology in humans |
| Q8DQ09 | Adhesion lipoprotein | Spr0906 | 1 | 0.95 | Yes | 1, 50MVKEVSGDLN59 | 6 | No |
| Q8DPQ2 | Pneumococcal histidine triad protein A | Spr1061 | 1 | 0.91 | No | - | - | - |
| Q8CZ52 | Endo-β-N-acetylglucosaminidase | Spr0440 | 2 | 0.95, 0.82 | Yes | - | 6 | No |
| Q8DQ98 | Hypothetical protein | Spr0777 | 1 | 0.12 | No | - | - | - |
| Q8CYB8 | Hypothetical protein | Spr1730 | 1 | 0.31 | No | - | - | - |

**SUPPLEMENTARY METHODS**

**Supplementary method 1**

**Detection of biotinylated proteins by dot blot.** In short, 2 μL S2 were spotted on an activated PVDF membrane. Membrane was air-dried and incubated with blocking solution (LI-COR, Biosciences) for 1 hour. Biotin-labeled proteins were detected by one hour incubation with 1:10,000 IRdye®800 CW Streptavidin in blocking solution, according to manufacturer´s instructions (LI-COR, Biosciences). Membrane was washed 3 times, two times with PBS containing 0.05% Tween20 for 5 minutes and once with PBS pH 7.4 for 5 minutes. Finally, signals were captured at 800 nm (Odyssey CLx, LI-COR bioscience). Biotinylated proteins of pneumococci and protein extract of human BMEC were used as input and negative control, respectively.

**Supplementary method 2**

**Capture of biotinylated proteins from cell extract.** Biotinylated proteins were recovered with NeutrAvidin agarose beads (Thermo Fisher Scientific) according to manufacturer´s instructions. In brief, cell lysate (S2) was incubated for 1 hour with 200 μL NeutrAvidin agarose beads previously equilibrated with PBS. Non-interacting proteins were removed by 5 washes each with 700 μL of PBS and centrifugation at 500 × g for 1 minute. Biotinylated proteins captured on the agarose beads were cleaved-off (elution) by incubating in 400 μL of 50 mM dithiothreitol (DTT) in PBS (pH 7.2). Proteins were recovered from the beads by centrifugation at 500 × g for 1 minute. Proteins in supernatant were dried (CentriVap concentrator, Labconco) for further MS analysis.

**Supplementary method 3**

**LC-MS SWATH analysis.** Protein digestion was performed with two-step in-solution digestion protocol according to the manufacturer’s instructions for Trypsin/LysC-Mix (Promega, USA). In short, eluate from streptavidin beads was mixed with 8 M urea in 50 mM Tris–HCl (pH 8.0) to a total volume of 10 μL. Proteins were reduced in 5 mM dithiothreitol for 30 min at 37 °C and alkylated with 15 mM iodoacetamide for 30 min at 25 °C. Trypsin/LysC mix and proteins in eluate were added in a ratio of 20:1 (w/w) and incubated for 4 hours at 37 °C. After incubation 50 mM Tris– HCl (pH 8.0) was added to bring the urea concentration to 1 mol/L. Mixture was again incubated for 8 h at 37 °C and then trifluoroacetic acid (TFA) was added to acidify the solution. Digested peptides were separated on a nano-LC (Ultimate 3000 Thermo-Fisher Scientific). Samples were concentrated and desalted with pre-column (Acclaim PepMap μ, Dionex) with 2% acetonitrile (ACN) and 0.05% TFA was used as a mobile phase. Peptides were then separated on a C18 column (25 cm Acclaim PepMap) with a flow rate of 250 nL/min. Gradient was started 4% of elution solution (80% ACN with 0.1% formic acid) and ended with 50% of elution solution. Elution time was kept 180 min. Peptides were analyzed with TripleTOF 5600+ (Sciex, USA). Information dependent data acquisition (IDA) was performed with survey scans ranging between 400–1600 m/z. The thirty most intense precursors with higher than 2 charge state with minimum 90 counts per second were selected for fragmentation. Ion scans were collected for MS2 in the range of 90–1700 m/z for 120 ms. Data was processed with ProteinPilot Software version 5.0 (Sciex, USA). For protein search, the UniProt database was restricted to *Streptococcus*. Mass tolerance in MS mode was set between 0.001 Da in MS and 0.01 Da in MS/MS mode for the search. The sample parameters were: trypsin digestion, cysteine alkylation - iodoacetamide, search effort - rapid ID. False discovery rate analysis (FDR) was performed using the integrated tools in ProteinPilot. Global false discovery rate was set to <1% on protein level. IDA identification results were used to create the SWATH ion library with the MS/MS with SWATH Acquisition MicroApp 2.0 in PeakView 2.2 (both Sciex, USA). Peptides were chosen based on a FDR rate <1%, excluding shared and modified peptides.

**Supplementary method 4**

**Selection and culture of clones for recombinant protein production.** Digested fragments were ligated into pQE-30-mCherry-GFP plasmid (in-house modified vector pQE-30 UA, Qiagen, Supplementary figure 4). Please note that in this vector mCherry serves as stuffer sequence, which is cut out during the digestion of vector with restriction enzymes. This vector allows fusion of 6X his tag at *N*-terminus of protein of interest and GFP tag at *C*-terminus. Ligation mix was purified using NucleoSpin (Macherey-Nagel, Germany) and transformed into *E. coli* M15 strain (Qiagen, Germany). Transformants were selected from LB agar plates (lysogeny broth, Sigma; supplemented with 2% bacteriological agar, 1% glucose, 25 µg/mL kanamycin and 50 µg/mL carbenicillin).

Clones were cultivated in Terrific broth (TB) (15 g/L tryptone, 30 g/L yeast extract, 12.5 g/L NaCl, 2.5 g/L MgCl_2_/MgSO_4_, 100 µL/L metal mix, 7.5 mL/L glycerol) supplemented with 1% glucose, 50 µg/mL carbenicillin and 25 µg/mL kanamycin until OD_600_= 6. Bacterial cells were pelleted (centrifugation at 6,000 × g for 10 minutes) and resuspended in fresh TB medium without glucose.

**Supplementary method 5**

In this study, we applied a combination of several free available web-based algorithms for the bioinformatic analysis of a set of identified proteins from proteomics for a selection of interacting proteins. Note that the tools used in this study could be combined in multiple forms, and here we studied one of those combinations for the selection and experimental validation of interacting proteins.

First, we assigned subcellular location to each identified protein and removed intracellular proteins with less chance to interact with host cells. To do so, we applied Locate P and Psortb to assign general categories for the subcellular location. LocateP (<http://www.cmbi.ru.nl/locatep-db/cgi-bin/locatepdb.py>) is a database containing subcellular location predictions of proteins expressed by several bacterial species. The information is displayed as a table and it is possible to extract specific data concerning to any subcellular localization. This algorithm is a user friendly platform, in which it is possible to retrieve elements by accession number (GI), gene name, protein length, or by information related with predictions (e.g. LocateP signal peptide possibility, predicted cleavage site, LocateP intracellular possibility, etc). Using this tool, we compared the columns containing the LocateP subcellular localization prediction and LocateP prediction by SwissProt Classification, to establish a subcellular prediction to each protein from our list. This platform offers the possibility to download data in tab-delimited format to manipulate it with other software like MS-Excel.

In parallel, we analyzed list of proteins with Psortb (<http://www.psort.org/psortb/>). This tool predicts subcellular location for one or more Gram-positive or Gram-negative bacterial sequences in FASTA format. In this study, FASTA file were downloaded from the UniProtKB repository using Retrieve/ID mapping (<http://www.uniprot.org/uploadlists/>). Psortb provides the localization scores for 5 sites (i.e. extracellular, cytoplasmic, cytoplasmic membrane, cell wall and unknown) and a final prediction score is given (if one site scores above the 7.5 cutoff). Prior to job submission, selection of organism and Gram stain are required. Output format must be selected in either short or long tab-delimited. Normal output displays results of each PSORTb’s analytical modules. Additional documentation for result interpretation is also provided ([http://www.psort.org/documentation/index.html#output](http://www.psort.org/documentation/index.html#output)). In this study, subcellular location was attributed to each protein after consensus of results obtained from the previously mentioned tools; and cytoplasmic proteins were not considered for further analysis.

Next, we applied a group of tools (named in this study as featured –based algorithms) to retrieve additional information such as presence and number of trans-membrane domains, signal peptide type I or II. TMHMM (<http://www.cbs.dtu.dk/services/TMHMM/>) was used to predict trans-membrane helix to those proteins predicted as membrane-attached proteins. Although it is available as a stand-alone software package, we run this server online. Submission is performed in FASTA format or pasting sequences in the displayed window. Output format is generated in three different formats, we recommend the extensive format with graphs in which the probability to have trans-membrane regions is showed along with the protein length (red color). Positions of outside (pink color) and inside amino acids (blue color) are also provided. Simultaneously, we analyzed protein sequences by HMMtop (<http://www.enzim.hu/hmmtop/>). This tool predicts trans-membrane helices and topology of proteins. In order to submit multiple protein sequences, go to “advanced”, select sequence format –FASTA, sequence type –single sequences and prediction type –reliable. Output format is sent as HTML or text file. Typical output file shows the protein sequence and assign to each amino acid the letters O, I or H that indicate outside, inside or helix, respectively. This tool provides position (i.e. number of amino acids) of trans-membrane helices. Final prediction for each protein was established based on a consensus between both predictors.

Signal peptides (SP) type I and II are short N terminal residue stretch present in the sequence of proteins exported out of the cytoplasm. SP-I is associated with secreted proteins and SP-II is present in case of lipoprotein. In this study, we predicted these SPs by SignalP (<http://www.cbs.dtu.dk/services/SignalP/>) and LipoP (<http://www.cbs.dtu.dk/services/LipoP/>). SignalP predicts the presence and location of signal peptide cleavage sites in amino acid sequences in prokaryotes and eukaryotes. Submission requires FASTA format, which is possible to paste in the input field or be uploaded as a FASTA file. For this study, settings were selected as follow: selection of organism - Gram-positive bacteria, output format - standard, D-cutoff values - default, and method - input sequences may include TM regions. This tool provides explanation of each parameter for a custom analysis. In this analysis, we focused exclusively on the presence or absence of SP which are indicated as SP= 'YES' or SP= 'NO', respectively. In regard to prediction of lipoproteins, presence of SP-II was evaluated by LipoP. Even though this tool also discriminates between other signal peptides and N-terminal membrane helices, we used it only for prediction of SP-II. LipoP output file contains the 'Best' prediction with an associated score and provide the predicted cleavage site (amino acid position and protein sequence) when SP is found. Based on these predictors, SP-I or SP-II were assigned.

Selection of surface-exposed proteins was performed by analyzing the results of all the tools mentioned above. Only surface proteins were subjected to further analysis (ontology analysis, literature review, protein interactive, antigenicity). Ontology annotations (GO) were retrieved using BLAST2GO (to install on local hard disk click here - <https://www.blast2go.com/blast2go-pro/download-b2g>), the software offers the possibility to import a single FASTA file containing multiple protein sequences in the study. Such file is recognized by selecting the option specific for protein sequences. It is also possible to skip duplicate entries. This software provides a user friendly interface and step by step visual guide. In first it is necessary to select the source to perform the BLAST, we recommend choosing NCBI database and default parameters. This step may be time-consuming depending on the number of sequences. Further steps involve the InterPRO analysis, GO mapping and KEGG pathways. Data is displayed as a flow graphs in which proteins are categorized. It is possible to create graphs by combining several levels of the displayed data. In this work, we considered annotations only when identity was higher than 95% in BLAST against the NCBI nr database.

We further performed interactive protein networks analysis by STRING (<https://string-db.org/>), this tool offers the possibility to identify proteins of a specific bacteria by uploading a FASTA file. STRING assigns protein names for each submitted protein sequence and the user must verify the proper protein assignation. After a protein list, STRING displays an interactive network for those interacting proteins. In settings it is possible to increase or decrease the level of confidence for analysis. In case of no interactive networks, we suggest to decrease this level to observe possible interactions or increase the number of interactors (proteins different to the submitted candidates) by including nodes in settings. In this study, we observed interactive networks of surface proteins of *S. pneumoniae* by using the default settings.

Finally, we evaluated antigenicity and immunogenicity in five protein candidates selected by the tools described above. ANTIGENpro ([https://scratch.proteomics.ics.uci.edu](https://scratch.proteomics.ics.uci.edu/)) is an pathogen-independent predictor of antigenicity based on sequence alignment. SCRATCH protein predictor contains several tools. In this study, we focused only in ANTIGENpro. During analysis, we manually pasted each protein sequence and identified by query. Outputs were received by email in a simple format containing the predicted probability of antigenicity (scale 0 to 1). In regard to the prediction of immunogenicity, we used Jenner Predict algorithm (<http://14.139.240.55/vaccine/validation.html>). In this on-line server it is necessary to go to job submission (link displayed in the left of the home page) and paste the protein sequence or upload the protein sequences in FASTA format. Then select Gram stain and submit. The output provides amino acid sequences identified as possible epitopes and its alignment with epitope sequence contained in the database (IEDB epitope ID). Predictions are shown in a table in which we can find the following categories: Pfam domain ID, Presence T-cell epitopes, Presence B cell epitopes, human homolog, conserve sequences in bacteria.

Combination of the bioinformatic tools briefly described here was successfully applied for the identification of interacting proteins of *S. pneumoniae* with brain microvascular endothelial cells. Even though we provide a short description of these tools, we strongly recommend the user to explores all these platforms to be familiar with the analysis.

**REFERENCES**

1. Choi, C. W. *et al.* Analysis of *Streptococcus* *pneumoniae* secreted antigens by immuno-proteomic approach. *Diagn. Microbiol. Infect. Dis.* **72,** 318–327 (2012).

2. Olaya-Abril, A. *et al.* Characterization of protective extracellular membrane-derived vesicles produced by *Streptococcus* *pneumoniae*. *J. Proteomics* **106,** 46–60 (2014).

3. Zakrzewicz, D. *et al.* Host-derived extracellular RNA promotes adhesion of *Streptococcus* *pneumoniae* to endothelial and epithelial cells. *Sci. Rep.* **6,** 37758 (2016).

4. Kallio, A. *et al.* Role of Pht proteins in attachment of *Streptococcus* *pneumoniae* to respiratory epithelial cells. *Infect. Immun.* **82,** 1683–1691 (2014).

5. Kloosterman, T. G. & Kuipers, O. P. Regulation of arginine acquisition and virulence gene expression in the human pathogen *Streptococcus* *pneumoniae* by transcription regulators ArgR1 and AhrC. *J. Biol. Chem.* **286,** 44594–44605 (2011).

6. Kilian, M., Mestecky, J. & Schrohenloher, R. E. Pathogenic species of the genus *Haemophilus* and *Streptococcus* *pneumoniae* produce immunoglobulin A1 protease. *Infect. Immun.* **26,** 143–149 (1979).

7. Yamaguchi, M. *et al.* Zinc metalloproteinase ZmpC suppresses experimental pneumococcal meningitis by inhibiting bacterial invasion of central nervous systems. *Virulence* **5594,** 00–00 (2017).

8. Beghetto, E. *et al.* Discovery of novel *Streptococcus pneumoniae* antigens by screening a whole-genome -display library. *FEMS Microbiol. Lett.* **262,** 14–21 (2006).

9. Turner, P. *et al.* Serum antibody responses to pneumococcal colonization in the first 2 years of life: results from an SE Asian longitudinal cohort study. *Clin. Microbiol. Infect.* **19,** E551-8 (2013).

10. Nieto, P. a, Riquelme, S. a, Riedel, C. a, Kalergis, A. M. & Bueno, S. M. Gene elements that regulate *Streptococcus pneumoniae* virulence and immunity evasion. *Curr. Gene Ther.* **13,** 51–64 (2013).

11. Papasergi, S. *et al.* Plasminogen- and fibronectin-binding protein B is involved in the adherence of *Streptococcus pneumoniae* to human epithelial cells. *J. Biol. Chem.* **285,** 7517–7524 (2010).

12. Hupp, S. *et al.* Astrocytic tissue remodeling by the meningitis neurotoxin pneumolysin facilitates pathogen tissue penetration and produces interstitial brain edema. *Glia* **60,** 137–146 (2012).

13. Rubins, J. B. & Janoff, E. N. Pneumolysin: a multifunctional pneumococcal virulence factor. *J. Lab. Clin. Med.* **131,** 21–27 (1998).

14. Jiménez-munguía, I. *et al.* Proteomics-driven design of a multiplex bead-based platform to assess natural IgG antibodies to pneumococcal protein antigens in children. *J. Proteomics* **126,** 228–233 (2015).

15. Ring, A., Weiser, J. N. & Tuomanen, E. I. Pneumococcal trafficking across the blood-brain barrier molecular analysis of a novel bidirectional pathway. *J. Clin. Invest.* **102,** 347–360 (1998).

16. Hammerschmidt, S. Adherence molecules of pathogenic pneumococci. *Curr. Opin. Microbiol.* **9,** 12–20 (2006).

17. Henriques-Normark, B. & Tuomanen, E. I. The pneumococcus: Epidemiology, microbiology, and pathogenesis. *Cold Spring Harb. Perspect. Med.* **3,** 1–15 (2013).

18. Ogunniyi, a. D., Woodrow, M. C., Poolman, J. T. & Paton, J. C. Protection against *Streptococcus pneumoniae* elicited by immunization with pneumolysin and CbpA. *Infect. Immun.* **69,** 5997–6003 (2001).

19. Ogunniyi, A. D. *et al.* Pneumococcal histidine triad proteins are regulated by the Zn2+-dependent repressor AdcR and inhibit complement deposition through the recruitment of complement factor H. *FASEB J.* **23,** 731–738 (2009).

20. Straume, D., Stams, G. A., Berg, K. H., Salehian, Z. & Havarstein, L. S. Identification of pneumococcal proteins that are functionally linked to penicillin-binding protein 2b (PBP2b). *Mol. Microbiol.* **103,** 99–116 (2017).

21. Goonetilleke, U. R., Scarborough, M., Ward, S. A. & Gordon, S. B. Proteomic analysis of cerebrospinal fluid in pneumococcal meningitis reveals potential biomarkers associated with survival. *J. Infect. Dis.* **202,** 542–50 (2010).

22. Heeg, C., Franken, C., van der Linden, M., Al-Lahham, A. & Reinert, R. R. Genetic diversity of pneumococcal surface protein A of *Streptococcus pneumoniae* meningitis in German children. *Vaccine* **25,** 1030–1035 (2007).

23. Oggioni, M. R. *et al.* Switch from planktonic to sessile life: A major event in pneumococcal pathogenesis. *Mol. Microbiol.* **61,** 1196–1210 (2006).

24. Suits, M. D. & Boraston, A. B. Structure of the *Streptococcus pneumoniae* Surface Protein and Adhesin PfbA. *PLoS One* **8,** 1–10 (2013).
