# Supplementary material for: Proteomic and bioinformatic pipeline to screen the ligands of S. pneumoniae interacting with human brain microvascular endothelial cells
Source: Sci Rep. 2018 Mar 27;8:5231. doi: 10.1038/s41598-018-23485-1 (PMC5869694; doi:10.1038/s41598-018-23485-1)
Supplement: Supplementary file 3 — Supplementary Dataset 2 [file 41598_2018_23485_MOESM3_ESM.docx]

**Proteomic and bioinformatic pipeline to screen the ligands of *S. pneumoniae* interacting with human brain microvascular endothelial cells**

**Irene Jiménez-Munguía^1^, Lucia Pulzova^1^, Evelina Kanova^1^, Zuzana Tomeckova^1^, Petra Majerova^2^, Katarina Bhide^1^, Lubos Comor^1^, Ivana Sirochmanova^1^, Andrej Kovac^2^ & Mangesh Bhide^1,2^*.**

**Supplementary Dataset 2: Recombinant fragments produced in this work.**

*In gray are highlighted the amino acid and nucleotide sequences included to make recombinant form of protein. Sequences highlighted with yellow color are primer sequences.*

**Protein name: Adhesion lipoprotein**

Uniprot accession number: Spr0906

aa sequence: 311

MVKELFMKKQNLFLVLLSVFLLCLGACGQKESQTGKGMKIVTSFYPIYAMVKEVSGDLNDVRMIQSSSGIHSFEPSANDIAAIYDADVFVYHSHTLESWAGSLDPNLKKSKVKVLEASEGMTLERVPGLEDVEAGDGVDEKTLYDPHTWLDPEKAGEEAQIIADKLSEVDSEHKETYQKNAQAFIKKAQELTKKFQPKFEKATQKTFVTQHTAFSYLAKRFGLNQLGIAGISPEQEPSPRQLTEIQEFVKTYKVKTIFTESNASSKVAETLVKSTGVGLKTLNPLESDPQNDKTYLENLEENMSILAEELK

Genbank accession number: AE007317.1: nt891426 to nt892361 (Gene length: 936 bp)

ATGGTTAAGGAGTTGTTCATGAAGAAACAAAATTTATTTTTAGTCCTGTTAAGTGTCTTTCTTTTATGCTTGGGGGCTTGTGGTCAAAAGGAAAGTCAGACAGGAAAGGGGATGAAAATTGTGACCAGTTTTTATCCTATCTACGCTATGGTTAAGGAAGTATCTGGTGACTTGAATGATGTTCGGATGATTCAGTCAAGTAGTGGTATTCACTCCTTTGAACCTTCGGCAAATGATATCGCAGCCATCTATGATGCAGATGTCTTTGTTTACCATTCTCATACACTCGAATCTTGGGCAGGAAGTCTGGATCCAAATCTAAAAAAATCCAAAGTGAAGGTCTTAGAGGCTTCTGAGGGAATGACCTTGGAACGTGTCCCTGGACTAGAGGATGTGGAAGCAGGGGATGGAGTTGATGAAAAAACGCTCTATGACCCTCACACATGGCTAGATCCTGAAAAAGCTGGAGAAGAAGCCCAAATTATCGCTGATAAACTTTCAGAGGTGGATAGTGAGCATAAAGAGACTTATCAAAAAAATGCGCAAGCCTTTATCAAAAAAGCTCAGGAATTGACTAAGAAATTCCAACCAAAATTTGAAAAAGCGACTCAGAAAACATTTGTAACACAACATACAGCCTTTTCTTATCTAGCGAAGAGATTTGGGCTTAATCAACTTGGTATTGCAGGTATCTCTCCTGAACAAGAACCAAGTCCACGACAACTAACAGAAATTCAGGAATTTGTTAAGACCTATAAGGTTAAAACGATTTTTACAGAAAGTAACGCTTCTTCAAAAGTAGCTGAAACTCTTGTCAAATCAACAGGTGTGGGTCTTAAAACTCTGAATCCTTTAGAGTCAGACCCACAAAATGACAAGACCTATTTAGAAAATCTTGAAGAAAATATGAGTATTCTAGCAGAAGAATTAAAGTGA

**Protein name: Hypothetical protein**

Uniprot accession number: Spr1730

aa sequence: 224

MKKKAFGIVLLVLAAWILLQGNFGIPSLDGKIWPLLGIVFFAYKSIESILRRHLTSAVFTGLLAIIIANYAYDLLPVTNHSLIWASILVVLGVGYLTHSSKFWNEKKWWYNGKKTVVTDKEVAFGSGTFYKQDQDLVDDQVEVAFGDAKIYYDNAEMLGDFATLNIEVAFGNATVYVPQHWRVDLKVETSFGAAKADAPVAPTSKTLTIRGDVAFGKLEIVYVK

Genbank accession number: AE007317.1: ntc1708366 to ntc1707692 (Gene length: 675 bp)

ATGAAAAAGAAAGCATTTGGTATTGTTTTATTGGTTTTAGCAGCTTGGATCTTGCTGCAAGGGAATTTTGGAATTCCTTCTTTGGATGGTAAAATATGGCCTTTACTAGGTATTGTTTTTTTTGCTTATAAGTCCATTGAGTCCATCCTTAGACGTCATCTCACTTCGGCAGTTTTTACAGGTTTACTGGCGATCATCATTGCAAATTACGCTTATGACTTGTTACCAGTTACCAATCATTCTCTTATTTGGGCTAGCATCTTGGTGGTACTTGGTGTTGGTTATCTGACGCATTCAAGTAAGTTCTGGAATGAAAAAAAATGGTGGTACAATGGGAAAAAAACAGTCGTCACGGATAAGGAAGTCGCTTTTGGTAGCGGGACCTTCTATAAGCAAGATCAAGATCTCGTAGATGACCAAGTGGAAGTCGCTTTTGGGGATGCTAAAATCTACTATGATAATGCAGAGATGCTAGGTGATTTTGCAACTTTAAATATTGAAGTGGCCTTCGGAAATGCAACCGTCTATGTTCCACAACACTGGCGTGTAGATTTGAAAGTAGAAACCTCCTTTGGTGCAGCTAAGGCTGACGCTCCTGTAGCCCCAACCAGCAAAACCTTGACTATCCGTGGAGATGTGGCTTTTGGGAAGTTGGAAATTGTCTACGTTAAATAA

**Protein name: Endo- β- N- acetylglucosaminidase**

Uniprot accession number: Spr0440

aa sequence: 1659

MKNPFFERRCRYSIRKLSVGACSLMIGAVLFAGPALAEETAVPENSGANTELVSGESEHSTNEADKQNEGEHARENKLEKAEGVATASETASPASNEAATTETAEAASAAKPEEKASEVVAETPSAEAKPKSDKETEAKPEATNQGDESKPAAEANKTEKEVQPDVPKNTEKTLKPKEIKFNSWEELLKWEPGAREDDAINRGSVVLASRRTGHLVNEKASKEAKVQALSNTNSKAKDHASVGGEEFKAYAFDYWQYLDSMVFWEGLVPTPDVIDAGHRNGVPVYGTLFFNWSNSIADQERFAEALKQDADGSFPIARKLVDMAKYYGYDGYFINQETTGDLVKPLGEKMRQFMLYSKEYAAKVNHPIKYSWYDAMTYNYGRYHQDGLGEYNYQFMQPEGDKVPADNFFANFNWDKAKNDYTIATANWIGRNPYDVFAGLELQQGGSYKTKVKWNDILDENGKLRLSLGLFAPDTITSLGKTGEDYHKNEDIFFTGYQGDPTGQKPGDKDWYGIANLVADRTPAVGNTFTTSFNTGHGKKWFVDGKVSKDSEWNYRSVSGVLPTWRWWQTSTGEKLRAEYDFTDAYNGGNSLKFSGDVAGKTDQDVRLYSTKLEVTEKTKLRVAHKGGKGSKVYMAFSTTPDYKFDDADAWKELTLSDNWTNEEFDLSSLAGKTIYAVKLFFEHEGAVKDYQFNLGQLTISDNHQEPQSPTSFSVVKQSLKNAQEAEAVVQFKGNKDADFYEVYEKDGDSWKLLTGSSSTTIYLPKVSRSASAQGTTQELKVVAVGKNGVRSEAATTTFDWGMTVKDTSLPKPLAENIVPGATVIDSTFPKTEGGEGIEGMLNGTITSLSDKWSSAQLSGSVDIRLTKSRTVVRWVMDHAGAGGESVNDGLMNTKDFDLYYKDADGEWKLAKEVRGNKAHVTDITLDKPITAQDWRLNVVTSDNGTPWKAIRIYNWKMYEKLDTESVNIPMAKAAARSLGNNKVQVGFADVPAGATITVYDNPNSQTPLATLKSEVGGDLASAPLDLTNQSGLLYYRTQLPGKEISNVLAVSVPKDDRRIKSVSLETGPKKTSYAEGEDLDLRGGVLRVQYEGGTEDELIRLTHAGVSVSGFDTHHKGEQNLTLQYLGQPVNANLSVTVTGQDEASPKTILGIEVSQEPKKDYLVGDSLDLSEGRFAVAYSNDTMEEHSFTDEGVEISGYDAQKTGRQTLTLRYQGHEVNFDVLVSPKAALNDEYLKQKLAEVEAAKNKVVYNFASPEVKEAFLKAIEAAEQVLKDHETSTQDQVNDRLNKLTEAHKALNGQEKFTEEKTELDRLTGEAQELLAAKPNHPSGSALAPLLEKNKALVEKVDLSPEELATAKQSLKDLVALLKEDKSAVFSDSKTGVEVHFSNKEKTVIKGLKVERVQASAEEKKYFAGEDAHVFEIEGLDEKGQDVDLSYASIVKIPIEKDKKVKKVFFLPEGKGAVELAFEQTDSHVIFTAPHFTHYAFVYESAEKPQPAKPAPQNKVLPKPTYQPASDQQKAPKLEVQEEKVAFHRQEHENTEMLVGEQRVIIQGRDGLLRHVFEVDEKGQRRLRSTEVIQEAIPEIVEIGTKVKTVPAVVATQEKPAQNTAVKSEEASKQLPNTGTADANEALIAGLASLGLASLALTLRRKREDKD

Genbank accession number: AE007317.1: nt441113 to nt446092 (Gene length: 4980 bp)

ATGAAGAATCCATTTTTTGAAAGACGTTGTCGTTACAGTATTCGTAAGTTATCAGTAGGAGCCTGCTCGCTGATGATTGGTGCTGTTTTATTTGCTGGTCCAGCCTTGGCTGAAGAAACTGCAGTTCCTGAAAATAGCGGAGCTAATACAGAGCTTGTTTCAGGAGAGAGTGAGCATTCGACCAATGAAGCTGATAAGCAGAATGAAGGGGAACATGCTAGAGAAAACAAGCTAGAAAAGGCAGAAGGAGTAGCGACAGCATCTGAAACTGCTTCGCCAGCAAGCAATGAAGCTGCAACTACTGAAACTGCAGAAGCAGCTAGCGCAGCTAAACCAGAGGAAAAAGCAAGTGAGGTGGTTGCAGAAACACCATCTGCAGAAGCAAAACCTAAGTCTGACAAGGAAACAGAAGCAAAGCCCGAAGCAACTAACCAAGGGGATGAGTCTAAGCCAGCAGCCGAAGCTAATAAGACTGAAAAAGAAGTCCAGCCAGATGTCCCTAAAAATACAGAAAAAACATTAAAACCAAAGGAAATCAAATTTAATTCTTGGGAAGAATTGTTAAAATGGGAACCAGGTGCTCGTGAAGATGATGCTATTAACCGTGGATCTGTTGTCCTCGCTTCACGTCGGACAGGTCATTTAGTCAATGAAAAAGCTAGCAAGGAAGCAAAAGTTCAAGCCTTATCAAACACCAATTCTAAAGCCAAAGACCATGCTTCTGTTGGTGGAGAAGAGTTCAAGGCCTATGCTTTTGACTATTGGCAATATCTAGATTCAATGGTCTTCTGGGAAGGTCTCGTACCAACTCCTGACGTTATTGATGCAGGTCACCGTAACGGGGTTCCTGTATACGGTACACTCTTCTTCAACTGGTCTAATAGTATTGCAGATCAAGAAAGATTTGCTGAAGCTTTGAAGCAAGACGCAGATGGTAGCTTCCCAATTGCCCGTAAATTGGTAGACATGGCCAAGTATTATGGCTATGATGGCTATTTCATCAACCAAGAAACAACTGGAGATTTGGTTAAACCTCTTGGAGAAAAGATGCGCCAGTTTATGCTCTATAGCAAGGAATATGCTGCTAAGGTAAACCATCCAATCAAGTATTCTTGGTACGATGCCATGACCTATAACTATGGACGTTACCATCAAGATGGTTTGGGAGAATACAACTACCAATTCATGCAACCAGAAGGAGATAAGGTTCCGGCAGATAACTTCTTTGCTAACTTTAACTGGGATAAGGCTAAAAATGATTACACTATTGCAACTGCCAACTGGATTGGTCGTAATCCTTATGATGTATTTGCAGGTTTGGAATTGCAACAGGGTGGTTCCTACAAGACAAAGGTTAAGTGGAATGACATTTTAGACGAAAATGGGAAATTGCGCCTTTCTCTTGGTTTATTTGCCCCAGATACCATTACAAGTTTAGGAAAAACTGGTGAAGATTATCATAAAAATGAAGATATCTTCTTTACAGGTTATCAAGGAGACCCTACTGGCCAAAAACCAGGTGACAAAGATTGGTATGGTATTGCTAACCTAGTTGCGGACCGTACGCCAGCGGTAGGTAATACTTTTACTACTTCTTTTAATACAGGTCATGGTAAAAAATGGTTCGTAGATGGTAAGGTTTCTAAGGATTCTGAGTGGAATTATCGTTCAGTATCAGGTGTTCTTCCAACATGGCGCTGGTGGCAGACTTCAACAGGGGAAAAACTTCGTGCAGAATATGATTTTACAGATGCCTATAATGGCGGAAATTCCCTTAAATTCTCTGGTGATGTAGCCGGTAAGACAGATCAGGATGTGAGACTTTATTCTACTAAGTTAGAAGTAACTGAGAAGACCAAACTTCGTGTTGCCCACAAGGGAGGAAAAGGTTCTAAAGTTTATATGGCATTCTCTACAACTCCAGACTACAAATTCGATGATGCAGATGCATGGAAAGAGCTAACCCTTTCTGACAACTGGACAAATGAAGAATTTGATCTCAGCTCACTAGCAGGTAAAACCATCTATGCAGTCAAACTATTTTTCGAGCATGAAGGTGCTGTAAAAGATTATCAGTTCAACCTAGGACAATTAACTATCTCGGACAATCACCAAGAGCCACAATCGCCGACAAGCTTTTCTGTAGTGAAACAATCTCTTAAAAATGCCCAAGAAGCGGAAGCAGTTGTGCAATTTAAAGGCAACAAGGATGCAGATTTCTATGAAGTTTATGAAAAAGATGGAGACAGCTGGAAATTACTAACTGGCTCATCTTCTACAACTATTTATCTACCAAAAGTTAGCCGCTCAGCAAGTGCTCAGGGTACAACTCAAGAACTGAAGGTTGTAGCAGTCGGTAAAAATGGAGTTCGTTCAGAAGCTGCAACCACAACCTTTGATTGGGGTATGACTGTAAAAGATACCAGCCTACCAAAACCACTAGCTGAAAATATCGTTCCAGGTGCAACAGTTATTGATAGTACTTTCCCTAAGACTGAAGGTGGAGAAGGTATTGAAGGTATGTTGAACGGTACCATTACTAGCTTGTCAGATAAATGGTCTTCAGCTCAGTTGAGTGGTAGTGTGGATATTCGTTTGACCAAGTCACGTACCGTTGTTAGATGGGTCATGGATCATGCAGGAGCTGGTGGTGAGTCTGTTAACGATGGCTTGATGAACACTAAAGACTTTGACCTTTATTATAAAGATGCAGATGGTGAGTGGAAGCTAGCTAAGGAAGTCCGTGGTAACAAAGCACACGTGACAGATATCACTCTTGATAAACCAATCACTGCTCAGGACTGGCGCTTGAATGTTGTCACTTCTGACAATGGAACTCCATGGAAGGCTATTCGTATCTATAACTGGAAAATGTATGAAAAGCTTGATACTGAGAGTGTCAATATTCCGATGGCCAAGGCTGCAGCCCGTTCTCTAGGCAATAACAAGGTACAAGTTGGCTTTGCAGATGTACCGGCTGGAGCAACTATTACCGTTTATGATAATCCAAATTCTCAAACTCCGCTCGCAACCTTGAAGAGCGAAGTTGGAGGAGACCTAGCAAGTGCACCATTGGATTTGACAAATCAATCTGGTCTTCTTTATTATCGTACCCAGTTGCCAGGCAAGGAAATTAGTAATGTCCTAGCAGTTTCCGTTCCAAAAGATGACAGAAGAATCAAGTCAGTCAGCCTAGAAACAGGACCTAAGAAAACAAGCTACGCCGAAGGGGAGGATTTGGACCTTAGAGGTGGTGTTCTTCGAGTTCAGTATGAAGGAGGAACTGAGGACGAACTCATTCGCCTAACTCACGCAGGTGTATCAGTATCAGGTTTTGATACGCATCATAAGGGAGAACAGAATCTTACTCTCCAATATTTGGGACAACCAGTAAATGCTAATTTGTCAGTGACTGTCACTGGCCAAGATGAAGCAAGTCCGAAAACTATTTTGGGAATTGAAGTAAGTCAGGAACCGAAAAAAGATTACCTAGTTGGTGATAGCTTAGACTTGTCTGAAGGACGCTTTGCAGTGGCTTATAGCAATGACACCATGGAAGAACATTCCTTTACTGATGAGGGAGTTGAAATTTCTGGTTACGATGCTCAAAAGACTGGTCGTCAAACCTTGACGCTTCGTTACCAAGGTCATGAAGTCAACTTTGATGTTTTGGTATCTCCAAAAGCAGCATTGAACGATGAGTACCTCAAACAAAAATTAGCAGAAGTTGAAGCTGCTAAGAACAAGGTGGTCTATAACTTTGCTTCACCAGAAGTAAAAGAAGCCTTCTTGAAAGCAATTGAAGCGGCCGAACAAGTGTTGAAAGACCATGAAACTAGCACCCAAGATCAAGTCAATGACCGACTTAATAAATTGACAGAAGCTCATAAAGCTCTGAATGGTCAAGAGAAATTTACGGAAGAAAAGACAGAGCTTGATCGCTTAACAGGTGAGGCTCAAGAACTCTTGGCTGCCAAACCAAACCATCCTTCAGGTTCTGCCCTAGCTCCGCTTCTTGAGAAAAACAAGGCCTTGGTTGAAAAAGTAGATTTGAGTCCAGAAGAGCTTGCAACAGCGAAACAGAGTCTAAAAGATCTGGTTGCTTTATTGAAAGAAGACAAGTCAGCAGTCTTTTCTGATAGTAAAACAGGTGTTGAAGTACACTTCTCAAATAAAGAGAAGACTGTCATCAAGGGTTTGAAAGTAGAGCGTGTTCAAGCAAGTGCTGAAGAGAAGAAATACTTTGCTGGAGAAGATGCTCATGTCTTTGAAATAGAAGGTTTGGATGAAAAAGGTCAAGATGTTGATCTCTCTTACGCTTCTATTGTGAAAATCCCAATTGAAAAAGATAAGAAAGTTAAGAAAGTATTTTTCTTACCTGAAGGCAAAGGGGCAGTAGAATTGGCTTTTGAACAAACGGATAGTCATGTTATCTTTACAGCACCACACTTTACTCATTATGCCTTTGTTTATGAATCTGCTGAAAAACCACAACCTGCTAAACCAGCACCACAAAACAAAGTCCTTCCAAAACCTACTTATCAACCGGCTTCTGATCAACAAAAGGCTCCTAAATTGGAAGTTCAAGAGGAAAAGGTTGCCTTTCATCGTCAAGAGCATGAAAATACTGAGATGCTAGTTGGGGAACAACGAGTCATCATACAGGGACGAGATGGACTGTTAAGACATGTCTTTGAAGTTGATGAAAAGGGTCAGCGTCGTCTTCGTTCAACAGAAGTCATCCAAGAAGCGATTCCAGAAATTGTTGAAATTGGAACAAAAGTAAAAACAGTACCAGCAGTAGTAGCTACACAGGAAAAACCAGCTCAAAATACAGCAGTTAAATCAGAAGAAGCAAGCAAACAATTGCCAAATACAGGAACAGCTGATGCTAATGAAGCCCTAATAGCAGGCTTAGCCAGCCTTGGTCTTGCTAGTTTAGCCTTGACCTTGAGACGGAAAAGAGAAGATAAAGATTAA

**Protein name: Hypothetical protein**

Uniprot accession number: Spr0777

aa sequence: 369

MFRRNKLFFWTTEILLLTIIFYLWRQMGSLINPFVSVLNTIMIPFLLGGFLYYLTNPIVTFLNKVCKLNRLLGILITLCTLVWGMVIGVVYLLPILINQLSSLIISSQTIYSRVQDLIIDLSNYPALQNLDVEATIQQLNLSYVDILQNILNSVSNSVGSVLSALISTVLILIMTPVFLVYFLLDGHKFLPMLERTILKRDRLHIAGLLKNLNATIARYISGVSIDAIIIGCLAYIGYSIIGLKYALVFAIFSGVANLIPYVGPSIGLIPMIIANIFTDPHRLLIAVIYMLVVQQVDGNILYPRIVGSVMKVHPITILVLLLLSSNIYGVVGMIVAVPTYSILKEISKFLSRLYENHKIMKERERELAK

Genbank accession number: AE007317.1: nt771852 to nt772961 (Gene length: 1110 bp)

ATGTTTCGTAGAAATAAATTATTTTTTTGGACCACAGAAATTTTACTCTTAACCATCATCTTTTACCTATGGAGACAGATGGGATCTTTGATTAACCCTTTTGTTAGCGTGCTTAATACAATTATGATTCCATTTTTATTAGGGGGCTTTCTTTATTATTTGACAAACCCTATTGTTACTTTCTTAAATAAAGTCTGTAAACTCAATCGTTTGCTTGGTATTTTAATTACCTTGTGTACTTTGGTCTGGGGAATGGTCATAGGTGTTGTCTATCTCTTACCTATTTTGATTAATCAGTTATCTAGTTTGATTATATCTAGTCAAACTATTTATAGTCGAGTACAAGACTTAATCATAGACTTATCTAATTATCCTGCGCTCCAGAATTTGGATGTAGAAGCTACAATTCAGCAGTTAAACTTATCCTATGTTGATATTCTTCAAAATATCCTAAATAGCGTATCAAATAGTGTGGGGAGCGTCTTGTCAGCTCTTATCAGTACTGTTTTGATTTTGATTATGACTCCAGTTTTTTTGGTTTATTTCTTATTAGATGGACATAAATTCTTGCCCATGCTTGAAAGAACGATTCTAAAGAGGGATCGCTTGCATATTGCAGGCTTATTAAAGAATTTAAATGCGACGATTGCTCGCTATATTAGTGGAGTTTCGATTGACGCAATCATTATAGGTTGTTTGGCTTATATTGGCTATAGTATTATTGGTTTAAAATATGCTTTAGTTTTTGCCATTTTTTCTGGTGTAGCCAATTTAATTCCTTATGTGGGGCCAAGTATTGGTTTGATTCCTATGATCATCGCAAATATATTCACTGATCCCCATAGACTGCTGATTGCAGTGATTTATATGCTTGTTGTTCAGCAGGTAGATGGCAATATCTTATATCCTCGAATTGTAGGAAGTGTTATGAAGGTTCATCCAATCACGATTTTAGTTTTACTTTTGTTGTCAAGCAATATCTATGGTGTAGTTGGAATGATTGTCGCAGTGCCAACCTATTCTATCTTGAAAGAAATTTCTAAGTTCTTATCCCGTTTGTATGAAAATCATAAAATAATGAAAGAACGAGAAAGAGAATTAGCTAAGTAA

**Protein name: Pneumococcal histidine triad protein A**

Uniprot accession number: Spr1061

aa sequence: 828

MQLEISNRKRVSMKINKKYLVGSAAALILSVCSYELGLYQARTVKENNRVSYIDGKQATQKTENLTPDEVSKREGINAEQIVIKITDQGYVTSHGDHYHYYNGKVPYDAIFSEELLMKDPNYKLKDEDIVNEVKGGYVIKVDGKYYVYLKDAAHADNVRTKEEINRQKQEHSQHREGGTPRNDGAVALARSQGRYTTDDGYIFNASDIIEDTGDAYIVPHGDHYHYIPKNELSASELAAAEAFLSGRGNLSNSRTYRRQNSDNTSRTNWVPSVSNPGTTNTNTSNNSNTNSQASQSNDIDSLLKQLYKLPLSQRHVESDGLVFDPAQITSRTARGVAVPHGDHYHFIPYSQMSELEERIARIIPLRYRSNHWVPDSRPEQPSPQPTPEPSPGPQPAPNLKIDSNSSLVSQLVRKVGEGYVFEEKGISRYVFAKDLPSETVKNLESKLSKQESVSHTLTAKKENVAPRDQEFYDKAYNLLTEAHKALFENKGRNSDFQALDKLLERLNDESTNKEKLVDDLLAFLAPITHPERLGKPNSQIEYTEDEVRIAQLADKYTTSDGYIFDEHDIISDEGDAYVTPHMGHSHWIGKDSLSDKEKVAAQAYTKEKGILPPSPDADVKANPTGDSAAAIYNRVKGEKRIPLVRLPYMVEHTVEVKNGNLIIPHKDHYHNIKFAWFDDHTYKAPNGYTLEDLFATIKYYVEHPDERPHSNDGWGNASEHVLGKKDHSEDPNKNFKADEEPVEETPAEPEVPQVETEKVEAQLKEAEVLLAKVTDSSLKANATETLAGLRNNLTLQIMDNNSIMAEAEKLLALLKGSNPSSVSKEKIN

Genbank accession number: AE007317.1: ntc1059416 to nt1056930 (Gene length: 2487 bp)

TTGCAACTAGAAATATCAAATAGAAAGAGAGTTTCGATGAAAATTAATAAGAAATACCTTGTTGGTTCTGCGGCAGCTTTGATTTTAAGTGTTTGTTCTTACGAGTTGGGACTGTATCAAGCTAGAACGGTTAAGGAAAATAATCGTGTTTCCTATATAGATGGAAAACAAGCGACGCAAAAAACGGAGAATTTGACTCCTGATGAGGTTAGCAAGCGTGAAGGAATCAATGCTGAGCAAATCGTCATCAAGATAACAGACCAAGGCTATGTCACTTCACATGGCGACCACTATCATTATTACAATGGTAAGGTTCCTTATGATGCGATTTTCAGTGAAGAATTACTCATGAAAGATCCAAACTATAAGCTAAAAGATGAGGATATTGTTAATGAGGTCAAGGGTGGATATGTTATCAAGGTAGATGGAAAATACTATGTTTACCTTAAGGATGCTGCCCACGCGGATAACGTCCGTACAAAAGAGGAAATCAATCGACAAAAACAAGAACATAGTCAACATCGTGAAGGTGGGACTCCAAGAAACGATGGTGCTGTTGCCTTGGCACGTTCGCAAGGACGCTATACTACAGATGATGGTTATATCTTTAATGCTTCTGATATCATAGAGGATACTGGTGATGCTTATATCGTTCCTCATGGAGATCATTACCATTACATTCCTAAGAATGAGTTATCAGCTAGCGAGTTGGCTGCTGCAGAAGCCTTCCTATCTGGTCGGGGAAATCTGTCAAATTCAAGAACCTATCGCCGACAAAATAGCGATAACACTTCAAGAACAAACTGGGTACCTTCTGTAAGCAATCCAGGAACTACAAATACTAACACAAGCAACAACAGCAACACTAACAGTCAAGCAAGTCAAAGTAATGACATTGATAGTCTCTTGAAACAGCTCTACAAACTGCCTTTGAGTCAACGACATGTAGAATCTGATGGCCTTGTTTTCGACCCAGCGCAAATCACAAGTCGAACAGCTAGAGGTGTTGCAGTGCCACACGGAGATCATTACCACTTCATCCCTTACTCTCAAATGTCTGAATTGGAAGAACGAATCGCTCGTATTATTCCCCTTCGTTATCGTTCAAACCATTGGGTACCAGATTCAAGGCCAGAACAACCAAGTCCACAACCGACTCCGGAACCTAGTCCAGGCCCGCAACCTGCACCAAATCTTAAAATAGACTCAAATTCTTCTTTGGTTAGTCAGCTGGTACGAAAAGTTGGGGAAGGATATGTATTCGAAGAAAAGGGCATCTCTCGTTATGTCTTTGCGAAAGATTTACCATCTGAAACTGTTAAAAATCTTGAAAGTAAGTTATCAAAACAAGAGAGTGTTTCACACACTTTAACTGCTAAAAAAGAAAATGTTGCTCCTCGTGACCAAGAATTTTATGATAAAGCATATAATCTGTTAACTGAGGCTCATAAAGCCTTGTTTGAAAATAAGGGTCGTAATTCTGATTTCCAAGCCTTAGACAAATTATTAGAACGCTTGAATGATGAATCGACTAATAAAGAAAAATTGGTAGATGATTTATTGGCATTCCTAGCACCAATTACCCATCCAGAGCGACTTGGCAAGCCAAATTCTCAAATTGAGTATACTGAAGACGAAGTTCGTATTGCTCAATTAGCTGATAAGTATACAACGTCAGATGGTTACATTTTTGATGAACATGATATAATCAGTGATGAAGGAGATGCATATGTAACGCCTCATATGGGCCATAGTCACTGGATTGGAAAAGACAGCCTTTCTGATAAGGAAAAAGTTGCAGCTCAAGCCTATACTAAAGAAAAAGGTATCCTACCTCCATCTCCAGACGCAGATGTTAAAGCAAATCCAACTGGAGATAGTGCAGCAGCTATTTACAATCGTGTGAAAGGGGAAAAACGAATTCCACTCGTTCGACTTCCATATATGGTTGAGCATACAGTTGAGGTTAAAAACGGTAATTTGATTATTCCTCATAAGGATCATTACCATAATATTAAATTTGCTTGGTTTGATGATCACACATACAAAGCTCCAAATGGCTATACCTTGGAAGATTTGTTTGCGACGATTAAGTACTACGTAGAACACCCTGACGAACGTCCACATTCTAATGATGGATGGGGCAATGCCAGTGAGCATGTGTTAGGCAAGAAAGACCACAGTGAAGATCCAAATAAGAACTTCAAAGCGGATGAAGAGCCAGTAGAGGAAACACCTGCTGAGCCAGAAGTCCCTCAAGTAGAGACTGAAAAAGTAGAAGCCCAACTCAAAGAAGCAGAAGTTTTGCTTGCGAAAGTAACGGATTCTAGTCTGAAAGCCAATGCAACAGAAACTCTAGCTGGTTTACGAAATAATTTGACTCTTCAAATTATGGATAACAATAGTATCATGGCAGAAGCAGAAAAATTACTTGCGTTGTTAAAAGGAAGTAATCCTTCATCTGTAAGTAAGGAAAAAATAAACTAA
